# DENV up-regulates the HMG-CoA reductase activity through the impairment of AMPK phosphorylation: A potential antiviral target

**DOI:** 10.1371/journal.ppat.1006257

**Published:** 2017-04-06

**Authors:** Rubén Soto-Acosta, Patricia Bautista-Carbajal, Margot Cervantes-Salazar, Antonio H. Angel-Ambrocio, Rosa M. del Angel

**Affiliations:** 1 Departmento de Infectómica y Patogénesis Molecular, CINVESTAV-IPN, México, D.F., México; 2 Department of Biochemistry and Molecular Biology, University of Texas Medical Branch, Galveston, TX, United States of America; Purdue University, UNITED STATES

## Abstract

Dengue is the most common mosquito-borne viral disease in humans. Changes of lipid-related metabolites in endoplasmic reticulum of dengue virus (DENV) infected cells have been associated with replicative complexes formation. Previously, we reported that DENV infection inhibits HMGCR phosphorylation generating a cholesterol-enriched cellular environment in order to favor viral replication. In this work, using enzymatic assays, ELISA, and WB we found a significant higher activity of HMGCR in DENV infected cells, associated with the inactivation of AMPK. AMPK activation by metformin declined the HMGCR activity suggesting that AMPK inactivation mediates the enhanced activity of HMGCR. A reduction on AMPK phosphorylation activity was observed in DENV infected cells at 12 and 24 hpi. HMGCR and cholesterol co-localized with viral proteins NS3, NS4A and E, suggesting a role for HMGCR and AMPK activity in the formation of DENV replicative complexes. Furthermore, metformin and lovastatin (HMGCR inhibitor) altered this co-localization as well as replicative complexes formation supporting that active HMGCR is required for replicative complexes formation. In agreement, metformin prompted a significant dose-dependent antiviral effect in DENV infected cells, while compound C (AMPK inhibitor) augmented the viral genome copies and the percentage of infected cells. The PP2A activity, the main modulating phosphatase of HMGCR, was not affected by DENV infection. These data demonstrate that the elevated activity of HMGCR observed in DENV infected cells is mediated through AMPK inhibition and not by increase in PP2A activity. Interestingly, the inhibition of this phosphatase showed an antiviral effect in an HMGCR-independent manner. These results suggest that DENV infection increases HMGCR activity through AMPK inactivation leading to higher cholesterol levels in endoplasmic reticulum necessary for replicative complexes formation. This work provides new information about the mechanisms involved in host lipid metabolism during DENV replicative cycle and identifies new potential antiviral targets for DENV replication.

## Author summary

DENV replicative complexes formation is associated with changes of lipid-related metabolites in endoplasmic reticulum, such as an increase in cholesterol synthesis. This increase correlates with a significant augment in the activity of HMGCoA reductase (the limiting enzyme in cholesterol synthesis), favoring a cholesterol-enriched cellular environment. The augment in the activity of the HMGCR observed in infected cells is caused by a decrease in the phosphorylation level of the HMGCR, associated with the inactivation of AMPK. In agreement, AMPK activation by metformin reduces HMGCR activity and affects viral replication. The role HMGCR and AMPK activity in DENV replicative complexes formation was confirmed by the co-localization of HMGCR and cholesterol with the viral proteins NS3, NS4A and E. Furthermore, metformin and lovastatin (HMGCR inhibitor) treatments altered this co-localization as well as replicative complexes formation supporting that active HMGCR is required for replicative complexes formation. The results show that during DENV infection, an increase in the HMGCR activity occurs through AMPK inactivation, leading to higher cholesterol levels in endoplasmic reticulum necessary for replicative complexes formation. This work provides new information about the mechanisms involved in host lipid metabolism during DENV replicative cycle and identifies potential new antiviral targets for DENV replication.

## Introduction

Dengue is one of the most relevant public health problems in tropical regions. The World Health Organization estimated that approximately 3.6 billion people are at risk for infection with dengue virus (DENV) in more than 100 countries, with an incidence of 390 million infections per year, of which 96 million of them are symptomatic [1]. Infection with any of the four DENV serotypes can be asymptomatic or present as a febrile illness called dengue fever. Occasionally, this febrile phase may evolve to more severe disease characterized by plasma leaking, fluid accumulation, respiratory distress, severe bleeding, and/or organ impairment. The most advanced dengue vaccine is the CYD-TDV vaccine (Sanofi Pasteur) that has been evaluated in two Phase III clinical trials in Asia and Latin America and approved in Mexico, Brazil and Philippines by the local health minister for use in endemic areas in these countries; however, the CYD-TDV is not fully efficacious [2]. This vaccine presented higher efficacy against DENV3 and DENV4 (71.6% and 76.9%, respectively) but lower efficacy for DENV1 and DENV2 (54.7 and 43%) [3].

DENV consists of a 50 nm particle containing a single positive-sense RNA genome of approximately 11 kb. The single open reading frame encodes for 10 viral proteins, 3 structural proteins: capsid (C), membrane (M) and envelope (E), and 7 nonstructural proteins: NS1, NS2A, NS2B, NS3, NS4A, NS4B, and NS5. Cellular lipids are important for several steps in the DENV replicative cycle [4,5]. Most of molecules described as receptors for DENV are present in cholesterol-rich complexes (lipid rafts) or are relocated to these structures after the interaction between receptor and viral particle [6–13]. Moreover, during a secondary infection by an antibody dependent mechanism (ADE), the Fc receptor (FcR) must be translocated to lipid rafts for DENV-antibody internalization and posterior infection. [14,15]. Zaitseva *et al*., (2010) demonstrated that DENV-fusion needs the presence of lipid cofactors such as anionic lipids (phosphatidylserine and bis(monoacylglycero) phosphate) in the endosome membrane in order for the envelope protein E to acquire a fusogenic conformation by after endosome acidification [16]. Upon viral attachment and endosomal fusion, the viral genome is translated by ribosomes at endoplasmic reticulum (ER). Significant evidence indicates that infection with (+) RNA viruses including DENV leads to the formation of membranous compartments called replication complexes, which operate as platforms where all required factors for viral RNA replication are concentrated [17]. The NS4A protein is the only viral factor directly associated with membrane rearrangement associated with DENV through its oligomerization [18]. Teo et al., 2013 reported that vimentin and its interaction with NS4A are important for the anchorage of replication complexes; also, vimentin reorganization and phosphorylation is fundamental to maintain the replication complexes structure[19,20]. The formation of replication complexes during DENV infection requires important modifications to host lipid metabolism [21–25]. Importantly, the reduction of DENV infection by inhibition of fatty acids and cholesterol biosynthesis pathways, as well as the relocation of enzymes involved in fatty acids metabolism to replication complexes and the increase of their activity, suggest that the supply of free fatty acids and cholesterol are important for membrane remodeling, which is necessary for DENV replication [26–30]. On the other hand, given the importance of lipid droplets for viral morphogenesis, cholesterol and lipids are also required for viral maturation [31,32]. Recently, our group reported an early increase in total cholesterol and lipid rafts formation after DENV infection of Huh7 cells. This effect correlated with an increased level of low density lipoprotein receptor (LDLr) on infected cells' surface at 1 hpi, and with a low phosphorylation of 3-hydroxy-3-methyl-glutaryl-CoA reductase (HMGCR, limiting enzyme of cholesterol synthesis is inhibited by phosphorylation at ser872) at 1, 9,12 and 18 hpi. This suggested that DENV infection increases intracellular cholesterol levels by triggering LDL particles uptake, and decreases the HMGCR phosphorylation causing an improved enzymatic activity [33]. Considering that the effect on HMGCR by DENV is longer than LDL uptake, and this protein is resident of endoplasmic reticulum, the dephosphorylation of HMGCR in DENV infected may be considered an important step for DENV replication cycle, nevertheless, mechanisms through which DENV infection modulates the HMGCR activity have not been elucidated.

The main upstream molecules that participate in HMGCR phosphorylation at ser872 are the 5' adenosine monophosphate(AMP)-activated protein kinase (AMPK), and the protein phosphatase 2A (PP2A). PP2A is the main serine/threonine phosphatase that dephosphorylates HMGCR raising its activity [34,35]. AMPK activity is regulated through phosphorylation in threonine 172 which in turn affect the intracellular lipid content mainly modulating the free fatty acids and cholesterol synthesis through direct phosphorylation of rate limiting enzymes, acetyl-CoA carboxylase (ACC) and HMGCR [36,37].

In this work, we evaluated the role of AMPK during DENV infection and demonstrated that DENV negatively regulates the AMPK activity at 24 hpi, which in turn drives an increase of HMGCR activity, resulting in cholesterol accumulation at replication sites. Pharmacological activation of AMPK produced a significant, dose-dependent anti-DENV effect. Interestingly, the treatment of infected cells with compound C (AMPK activator) produced a slight increase in the infection process highlighting AMPK as a new potential antiviral target.

## Results

### DENV infection down-regulates AMPK phosphorylation

**Fig 1:**
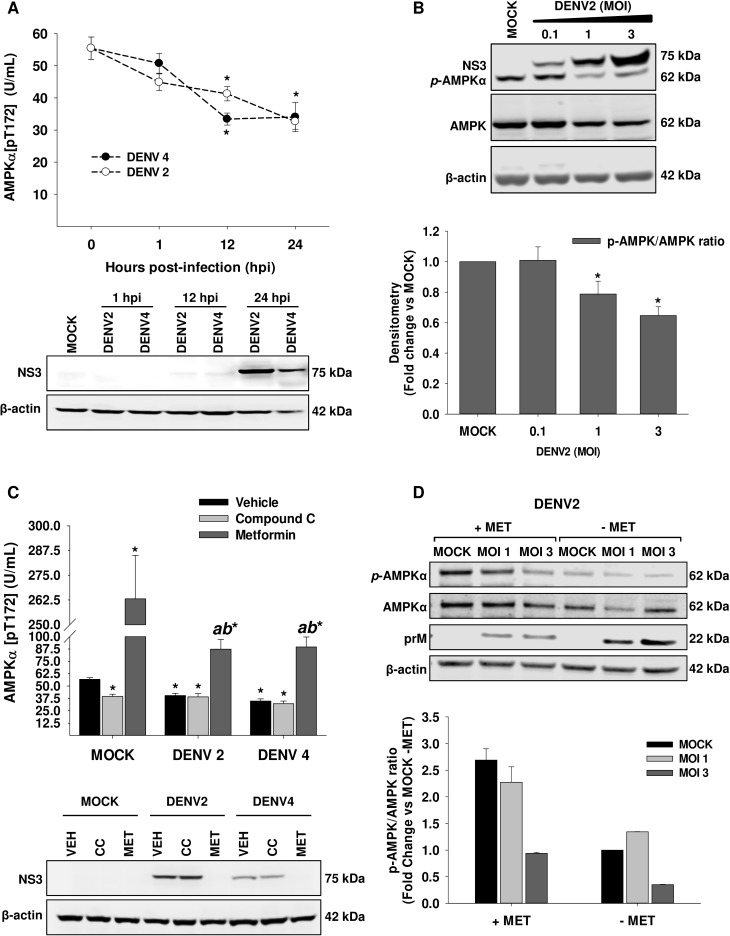
DENV infection down-regulates AMPK activity. In **A,** The AMPK activity, depicted as phosphorylation levels at Thr-172, was evaluated in Huh7 cells infected with DENV 2/4 (MOI 3) at 1, 12, and 24 hpi by ELISA, and NS3 viral protein levels *(A*, *lower panel)* were determined as infection test. AMPK activity was expressed as U/mL. *p<0.*05* compared to mock infected cells (0 hpi). Data are means ± standard error (S.E) of *n = 3* independent experiments realized by duplicate. **(B)** The levels of AMPK phosphorylated, AMPK total, and NS3 viral protein were analyzed by western blot in whole cell lysates obtained from Huh7 cells infected with DENV2 (MOI 0.1, 1 and 3) for 24 h. Graph represents the relative quantification of *p*AMPK respect to AMPK total protein. The *p*AMPK and total AMPK densitometry values were normalized with β-actin and pAMPK/AMPK ratios were calculated, Ratios are represented with respect to the indicated control. *p<0.*05* compared to mock infected cells. Data are means ± standard error (S.E) of *n = 4* independent experiments. **(C)** The AMPK activity and NS3 viral protein levels *(C*, *lower panel)* were determined in Mock or DENV 2/4 infected Huh7 cells treated with DMSO 0.5% (vehicle, VEH), 10 mM Metformin (MET, AMPK activator) or 10 μM Compound C (CC, AMPK inhibitor) for 24 h. * *p<0*.*05* compared to mock VEH-treated cells, ^***ab***^ *p<0*.*05* compared to mock MET-treated cells. Data are means ± standard error (S.E) of *n = 3* independent experiments realized by duplicate. (**D)** The levels of AMPK phosphorylated, AMPK total, and prM viral protein were analyzed by western blot in whole cell lysates obtained from Mock or DENV2 Huh7 infected cells (MOI 1 and 3) in the presence or absence of 10 mM metformin (MET) for 24h. Graph represents pAMPK/AMPK ratios normalized with respect to Mock infected cells with no MET treatment. pAMPK/AMPK ratios were obtained adjusting each protein with β-actin.

The reduction of the HMGCR phosphorylation is related with an increased activity [38]. Previously, our group reported that DENV infection decreases the phosphorylated levels of HMGCR at early time post-infection in Huh7 cells [33], which suggest that during DENV infection this enzyme is activated. The main upstream kinase involved in HMGCR phosphorylation is the 5' adenosine AMP-activated protein (AMPK). AMPK activity is modulated through the phosphorylation in the threonine 172. To evaluate if DENV infection modulates the AMPK activity, the phosphorylation levels of AMPK at Thr-172 were evaluated by ELISA in mock and DENV infected cells (MOI 3) at different hour post-infection (hpi). The time course of AMPK activity in DENV infected cells (serotype 2 and 4) showed a reduction of AMPK phosphorylation (AMPKαT172) at 12 and 24 hpi compared to mock cells **(Fig 1A)**. NS3 viral protein was evaluated as evidence of infection **(Fig 1A, lower panel)**. The reduction of AMPKα phosphorylation induced by DENV at 24 hpi was also observed by western blot using antibodies directed against AMPKα phosphorylated form and total AMPKα. A MOI-dependent reduction of AMPK phosphorylation was observed in DENV2 infected cells at MOI of 1 and 3. **(Fig 1B)**. Next, we confirmed these results using a pharmacological approach. Mock and DENV 2/4 infected cells were treated with DMSO 0.5% (vehicle, VEH), compound C (CC, AMPK inhibitor), and metformin (MET, AMPK activator) for 24h, and AMPK phosphorylation was analyzed by ELISA **(Fig 1C)**. As expected, under control condition (mock cells) the CC-treatment inhibited the AMPK activity (39.57 ± 2.21 U/mL), while MET treatment enhanced the AMPK activation (263.16 ± 21.9 U/mL) compared to the mock VEH-treated cells (56.76±1.61 U/mL). Similarly, AMPK activity decayed in DENV 2/4 infected cells treated with VEH at 24 hpi (DENV2: 40.35 ± 2.3 U/mL, and DENV4: 34.9 ± 2.14 U/mL) respect to mock VEH-treated cells. This activity was similar to the observed with CC-treatment in mock cells (39.57 ± 2.21 U/mL). However, CC-treatment of infected cells did not cause a further reduction in the AMPK activity (DENV2: 38.97 ± 3.45, and DENV4: 32.11 ± 2.70). In contrast, MET-treatment enhanced the AMPK activity in DENV 2/4 infected cells (DENV2: 87.28 ± 9.85, and DENV4: 89.48 ± 10.21) compared to mock VEH-treated cells, but this increase was significantly lower (up to 2-fold) than the one observed in mock MET-treated cells **(Fig 1C)**. Levels of NS3 viral protein were determined as infection evidence. Interestingly, MET-treated infected cells reduced the NS3 levels compared to VEH-treated infected cells **(Fig 1C, lower panel)**. The AMPK phosphorylation was confirmed by western blot in mock or DENV2 infected cells treated with metformin for 24 h **(Fig 1D)**. Likewise, the prM viral protein levels were reduced by metformin treatment **(Fig 1D)**. These results demonstrate that DENV infection decreased the AMPK activity and suggest that the activation of AMPK induces a strong anti-DENV effect.

### The increased activity of HMGCR in DENV infected cells is mediated by AMPK inhibition

**Fig 2:**
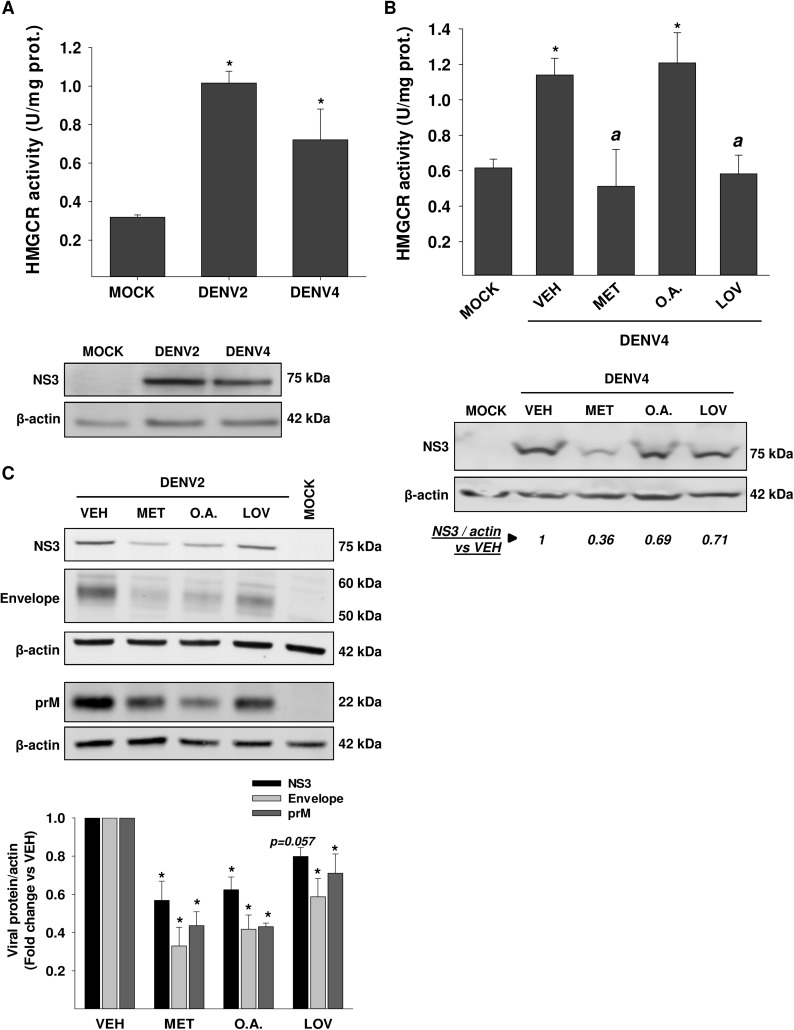
HMGCR activity is increased during DENV infection through down-regulation of AMPK. In **A,** The enzymatic activity of HMGCR was evaluated in Huh7 cells infected with Mock or DENV 2/4 (serotype 2 or 4, MOI 3) at 24 hours post-infection (hpi). HMGCR activity was expressed as U/mg protein. *p<0.*05* compared to mock infected cells. From same cell lysates, levels of NS3 viral protein *(A*, *lower panel)* were determined by WB as infection test. **(B)** DENV4 infected Huh7 cells treated with DMSO 0.5% (vehicle, VEH), 10 mM metformin (MET, AMPK activator), 50 μM lovastatin or 10 nM Okadaic acid (O.A, PP2A inhibitor) for 24 hpi were assayed for HMGCR activity and NS3 viral protein levels *(B*, *lower panel)*. *p<0.*05* compared to mock infected cells, ^***a***^ *p<0*.*05* compared to DENV4 VEH-treated cells. Relative quantification of *NS3* levels (*numbers in italics*) was normalized to β-actin and represented with respect to the indicated control. (**C)** The levels of NS3, E (envelope), and prM viral proteins were analyzed by western blot in whole cell lysates obtained from Mock or DENV2 Huh7 infected cells (MOI 3) treated with DMSO 0.5% (vehicle, VEH), 10 mM metformin (MET, AMPK activator), 10 nM Okadaic acid (O.A, PP2A inhibitor), and 50 μM Lovastatin (LOV, HMGCR inhibitor) for 24 h. Graph represents the relative quantification each protein normalized to β-actin and represented with respect to the indicated control (VEH). All data are means ± standard error (S.E) of *n = 3* independent experiments.

Next, we evaluated whether the increase of HMGCR activity is modulated through the AMPK inhibition induced by DENV. First, using an enzymatic assay, we confirmed that DENV 2/4 infection increases the HMGCR activity at 24 hpi (DENV2: 1.02 ± 0.0625 U/mg protein, and DENV4: 0.72 ± 0.16 U/mg protein) respect to mock infected cells (0.32 ± 0.01 U/mg protein) **(Fig 2A)**. NS3 was detected as evidence of infection **(Fig 2A, lower panel).** Then, the increase of HMGCR activity was evaluated in DENV infected cells (24 h) treated with DMSO 0.5% (VEH, vehicle), metformin (MET, AMPK activator), and lovastatin (LOV, HMGCR inhibitor) as a positive control. DENV infection increased the HMGCR activity in VEH-treated cells (1.14±0.09 U/mg protein) compared to mock infected cells (0.62±0.05 U/mg protein) **(Fig 2B)**. This activity was inhibited by metformin (0.51±0.21 U/mg protein) at the level observed in mock infected cells **(Fig 2B)**. Similar inhibition was observed with the competitive inhibitor of HMGCR lovastatin (0.58±0.11 U/mg protein) **(Fig 2B)**. These results suggest that the increased HMGCR activity triggered by DENV infection is mediated by AMPK. While AMPK is involved in the phosphorylation of HMGCR, the phosphatase PP2A activates HMGCR directly through its dephosphorylation [39]. For this reason, DENV-infected cells were treated with okadaic acid (O.A), a PP2A inhibitor, at a concentration that decreased PP2A activity (10 nM) (shown below); however O.A. did not have any effect on the HMGCR activity (1.21 ± 0.17 U/mg protein) respect to DENV VEH-treated cells, suggesting that PP2A is not involved in the up-regulation of HMGCR activity induced by DENV. In all conditions, the levels of NS3 viral protein were analyzed by western blot as infection evidence. A highest reduction in the levels of NS3 was observed with MET treatment **(Fig 2B, lower panel)**. This effect was reproducible in DENV2 infected cells treated with metformin, okadaic acid and lovastatin **(Fig 2C)**. A decrease in levels of non-structural protein NS3, and structural proteins E and prM was observed **(Fig 2C),** suggesting that pharmacological activation of AMPK by MET and OA treatment could have an antiviral effect. As expected, we observed a reduction of viral proteins induced by lovastatin **(Fig 2C)**. Together, our results show that AMPK is involved in the up-regulation of HMGCR activity by DENV. Furthermore, MET reduced the amount of viral proteins more than lovastatin, suggesting that AMPK activation could alter important pathways for DENV replication in addition to the inhibition of the HMGCR activity.

### HMGCR co-localizes with NS4A in replicative complexes and this process is modulated by the HMGCR activation dependent of AMPK

**Fig 3:**
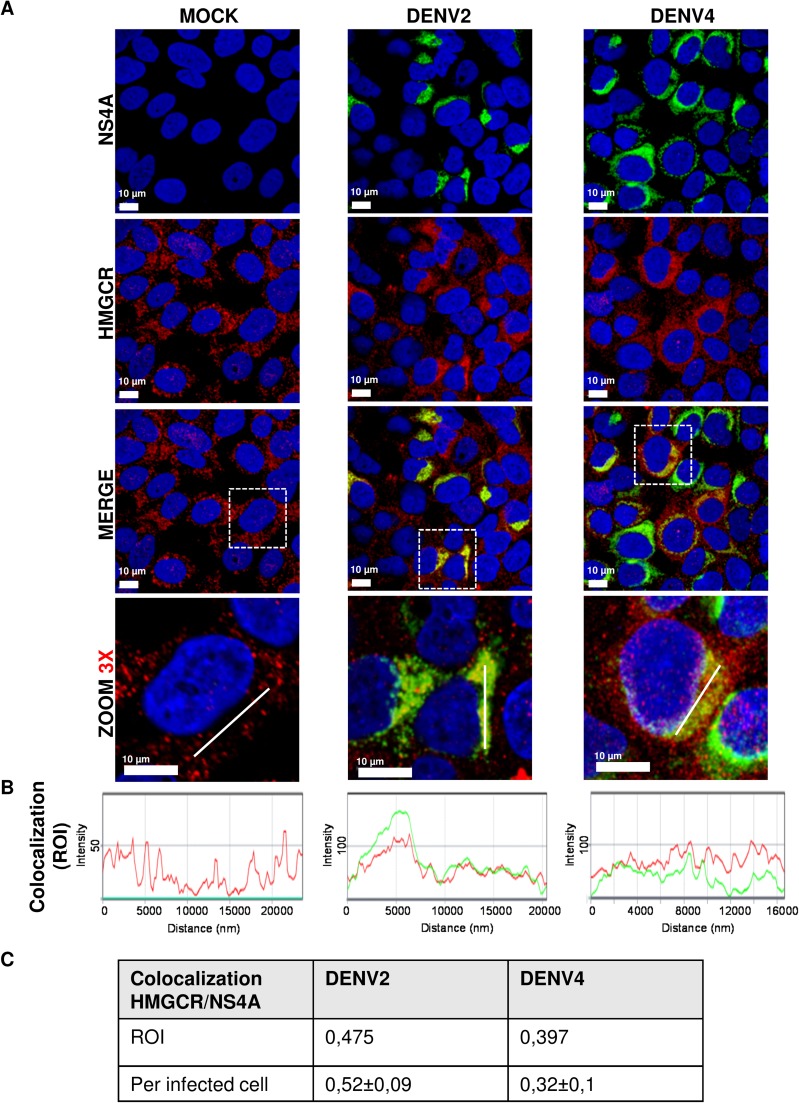
Intracellular distribution of HMGCR and NS4A viral protein during DENV infection. **(A)**The distribution of the NS4A ***(green)*,** a viral protein present at DENV-replication complexes, and the HMGCR ***(red)*,** a cellular ER-resident protein, was evaluated by confocal microscopy in Huh7 cells infected (MOI 3) with DENV2 and DENV4 at 24 hpi. Nuclei were stained with Hoechst ***(blue)***. Scale bar 10 μm. White dashed boxes are depicting the zoom area. **(B)** Histograms represent the fluorescence intensity for NS4A and HMGCR in determined area (white continuous line) demonstrating the correlation between two signals. In all infected cells, HMGCR colocalized with NS4A, however, the optical cut does not allow us to clearly observed this colocalization. **(C)**The table indicates HMGCR/NS4A colocalization values for region of interest (ROI, white dashed boxes) and colocalization per infected cell expressed as mean ± S.E. of 52 DENV2 infected cells and 47 DENV4 infected cells from three independent images.

Several reports have shown the antiviral effect of several cholesterol-lowering drugs that inhibit HMGCR activity, suggesting an important role for cholesterol metabolism during DENV replication [27,28,40]. In fact, DENV infection can modulate lipid metabolism and recruit to DENV's replication complexes the enzyme Fatty Acid Synthase (FAS), involved on free fatty acid anabolism, increasing its activity [22,29,30]. Since that HMGCR is an ER-resident protein and its raised activity during DENV infection is mediated by down-modulation of AMPK (as was observed at Fig 1), we hypothesize that HMGCR could be present in DENV replication sites and its elevated activity is required in order to build and maintain the replication complexes structure. For this reason the first step was to analyze the presence of HMGCR in replication complexes by confocal microscopy. Replication complexes were identified by its immunoreactivity for NS4A protein, a viral protein that is present [41] and elicits the membrane curvature needed to the replicative complexes formation [20,42]. The distribution patterns of NS4 and HMGCR correlated in DENV2/4 infected cells at 24 hpi resulting in a HMGCR/NS4A colocalization ratio of 0,52 ± 0,09 per DENV2 infected cell and 0,32 ± 0,1 per DENV4 infected cell (**Fig 3**), which suggest that both proteins are present in a close proximity. We corroborated the proximity of HMGCR (10 nm gold particle) with the NS4A protein (30 nm gold particle) by transmission electron microscopy in DENV infected cells at 24 hpi (**S1 Fig.**), which demonstrated that HMGCR co-localized with NS4A in DENV’s replication complexes.

**Fig 4:**
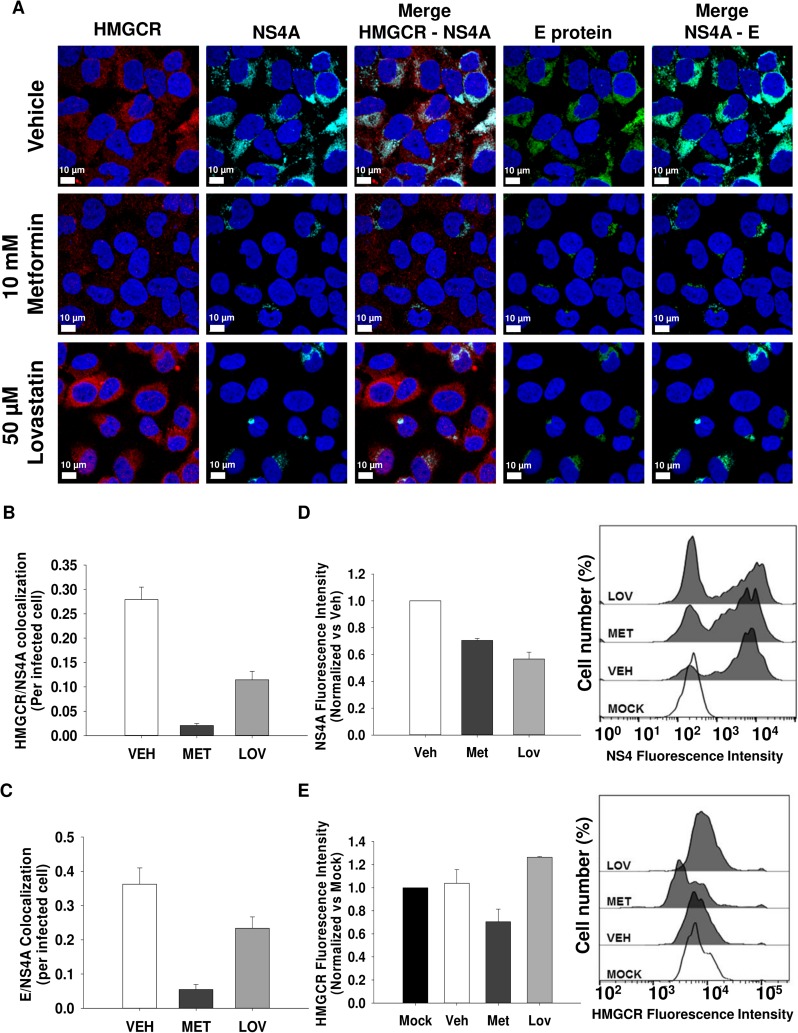
Activated HMGCR is required for the formation of DENV-replication complexes and the maintenance of its architecture. The distribution of HMGCR and components of viral replication complexes (NS4A and E viral proteins) was evaluated by confocal microscopy in Huh7 cells infected with DENV2 (MOI 3) and treated with DMSO 0.5% (vehicle), 10 mM Metformin or 50 μM lovastatin (HMGCR inhibitor) for 24h. The integrity of replication complexes is depicted as the co-localization between NS4A and E proteins. In **A** is indicated the distribution of HMGCR **(red)**, NS4A **(light blue)**, and E protein **(green)** as well as the colocalization per infected cell of NS4A/HMGCR **(B)** and NS4A/E **(C)** represented by mean ± S.E of the colocalization of 60 infected cells per condition. **D** and **E** represent the mean fluorescence intensity of NS4A protein **(D)** and HMGCR **(E)** analyzed by flow cytometry. Graphs represent the mean fluorescence intensity ± S.E of three independent experiments, the histograms indicate the fluorescence intensity of a representative experiment.

Next, to determine if the increased HMGCR activity mediated by AMPK inhibition during DENV infection plays a role in the replicative complex structure, DENV infected cells were treated with DMSO 0.5% (vehicle), metformin (AMPK activator) or lovastatin (HMGCR inhibitor) for 24h, and the HMGCR co-localization with the viral proteins NS4A and E, as well as replicative complex integrity were analyzed by confocal microscopy. In infected vehicle-treated cells, HMGCR showed co-localization with NS4A (co-localization = 0.28 ± 0.026, **Fig 4B**) as well as with NS3 (co-localization = 0.36 ± 0.04, **S2A and S2B Fig**). The replicative complexes integrity depicted as the co-localization between NS4A and E viral proteins or NS3 and E, was maintained in infected vehicle-treated cells showing a co-localization of 0.36 ± 0.05 between NS4A and E (**Fig 4C**) and a colocalization of 0.41 ± 0.06 between NS3 and E (**S2A and S2C Fig**)

In contrast, the treatment with MET altered the distribution of HMGCR, NS4A (**Fig 4A**), and NS3 protein (**S2A and S2B Fig)**. Likewise, the co-localization between HMGCR and viral proteins NS4A (**Fig 4A and 4B**) or NS3 (**S2A and S2B Fig)** was disrupted in DENV MET-treated cells, and the replicative complexes integrity as well (NS4A and E co-localization in **Fig 4A and 4C**, or NS3 and E co-localization in **S2A and S2C Fig**). This effect could be due to that AMPK activation reduced the HMGCR protein levels (**Fig 4,**MET-treated cells). However, the lovastatin-treatment, which resulted in the same effect than MET-treatment, elicits a slight increase in the HMGCR protein levels (**Fig 4** lovastatin-treated cells), which demonstrates that replication complexes formation needs the HMGCR activation. Additionally, the reduction of viral proteins (NS4A and NS3) observed in response to the treatment with MET and lovastatin was corroborated measuring the viral proteins by flow cytometry demonstrating that metformin and lovastatin promoted a decrease of NS4A (**Fig 4D**) and NS3 proteins levels (**S2D Fig**). Interestingly, differences in HMGCR expression in response to treatment with MET and lovastatin were observed in the confocal images. These differences in the expression levels of HMGCR were analyzed by flow cytometry in infected cells treated with vehicle, MET or lovastatin. The results indicate that MET decreases HMGCR expression **(Fig 4E)** which is consistent with the results obtained by Madsen et al., 2015 whose demonstrated that MET decreases HMGCR expression and other genes related with lipid metabolism by transcriptional suppression of the steroid receptor coactivator 2 (SRC-2)[43] In contrast, lovastatin induced an increase in the amount of HMGCR **(Fig 4E)** confirming previous reports in hepatic and neuron cell lines [44]. This effect can be explained by the regulatory feedback induced by the reduction of intracellular cholesterol levels, which trigger HMGCR transcription [45].

**Fig 5:**
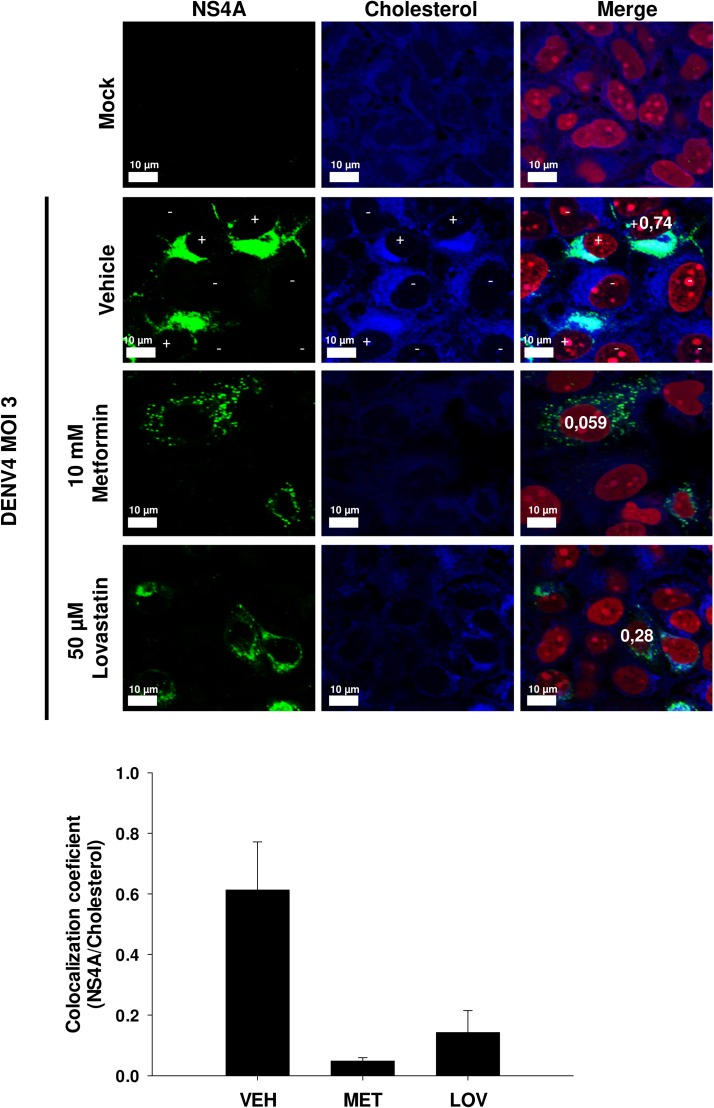
DENV infection stimulates the intracellular cholesterol accumulation at replicative complexes through the activation of HMGCR. The distribution of intracellular cholesterol levels stained with filipin III complex ***(blue)*,** and its co-localization with the viral protein NS4A ***(green)*** were evaluated by confocal microscopy in Huh7 cells non-infected, infected with DENV4 and treated with DMSO 0.5% (vehicle), 10 mM metformin or 50 μM lovastatin (HMGCR inhibitor) for 24 h. DENV4 infected cells are marked with **(+),** and non-infected cells marked with **(-)**. Nuclei were stained with propidium iodide ***(red)***. Numbers inserted in images indicate the co-localization index between cholesterol and NS4A for that specific infected cell. Graph represents NS4A and cholesterol colocalization values as mean ± S.E of 50 infected cells analyzed from 3 independent experiments. Scale bar 10 μm. Images correspond to one representative experiment.

Previously, we reported the increase in total cholesterol levels in DENV infected cells[33]. The high activity of HMGCR in replicative complexes from infected cells suggests that DENV infection could lead accumulation of intracellular cholesterol through HMGCR activation in order to encourage the formation and maintenance of DENV-replicative complexes. To investigate this possibility, intracellular cholesterol levels were stained with filipin III complex, and its accumulation/distribution and co-localization with the NS4A viral protein were analyzed by confocal microscopy at 24 hpi in infected cells treated with DMSO 0.5% (vehicle), metformin and lovastatin. In DENV infected cells treated with the vehicle (**Fig 5**, infected cells **marked with “+”**), cholesterol accumulation/distribution was observed in the same regions where NS4A was present (co-localization = 0.61) demonstrating that there is cholesterol accumulated in DENV-replication complexes, in contrast to the non-infected cells (Mock cells) and non-infected cells present in the same image (**cells marked with “-”**). Conversely, when cells were treated with an AMPK activator (metformin) or with a HMGCR competitive inhibitor (lovastatin), the accumulation of cholesterol and NS4A was lowered, and their distribution was altered (**Fig 5**), demonstrating that cholesterol accumulation is an important event for the DENV-replicative complexes formation, which is mediated by the HMGCR activity rise induced via AMPK inactivation. These data are consistent with the total cholesterol increment in DENV-infected Huh7 cells reported previously by our group [33]. Together, our results suggest that inhibition of AMPK activity caused during DENV infection could enhance the activity of HMGCR, which is present in DENV-replicative complexes stimulating the cholesterol accumulation.

Treatment with MET and lovastatin decreased the amount of structural and non-structural viral protein including NS4A and caused an inhibition of RC formation. Moreover, MET also induced a reduction in the levels of dsRNA (**S6 Fig**), supporting the idea that MET inhibits viral replication. However, it is not clear if the absence of cholesterol inhibited RC formation or the reduction in NS proteins levels in treated cells inhibited RC formation. For this reason, since we know that NS4A is essential for the RC formation because it is able to induce membrane curvature in this organelle when is expressed alone [41,42,46], the effect of the AMPK activation and the reduction of HMGCR activity in the distribution of NS4A was evaluated. Thus, Huh7 cells were transfected with a plasmid encoding DENV NS4A-eGFP [46] and were treated with vehicle (VEH) or with MET, ethyl 2-[2, 3, 4-trimethoxy-6-(1-octanoyl)phenyl]acetate (TMPA, indirectly activates AMPK through the regulation of the Liver Kinase B1 localization[47]), A-769662 (directly activates AMPK mimicking the effect of AMP [48]) and lovastatin. The distribution pattern of NS4A was analyzed by fluorescence microscopy. In non-treated cells (VEH) the distribution of NS4A-eGFP was observed compact in the perinuclear region. This distribution is consistent with the distribution of the RC complexes during DENV infection. However, treatments with the AMPK activators and lovastatin induced a disperse distribution of NS4A, but still in the ER, suggesting that the absence of cholesterol induced by the drugs inhibited the ability of NS4A to induce its compact distribution in the perinuclear region (**S3 Fig**). Treatments did not change distribution of eGFP in cells transfected with plasmid encoding just eGFP (**S4 Fig**) used as a control. These results are consistent with the idea that the reduction in cholesterol levels induced by the drugs, altered the ability of NS4A to be associated and build the RC. However, in this case the effect observed was not as drastic as the effect observed during DENV infection. This observation could be related with the fact that under transfection, a higher expression of the protein is observed while in infected cells, the inhibition in RC formation induces a reduction in the synthesis of viral proteins, among them NS4A, making more drastic the effect of the reduction in cholesterol levels.

### AMPK-activation mediated by metformin inhibits DENV infection

**Fig 6:**
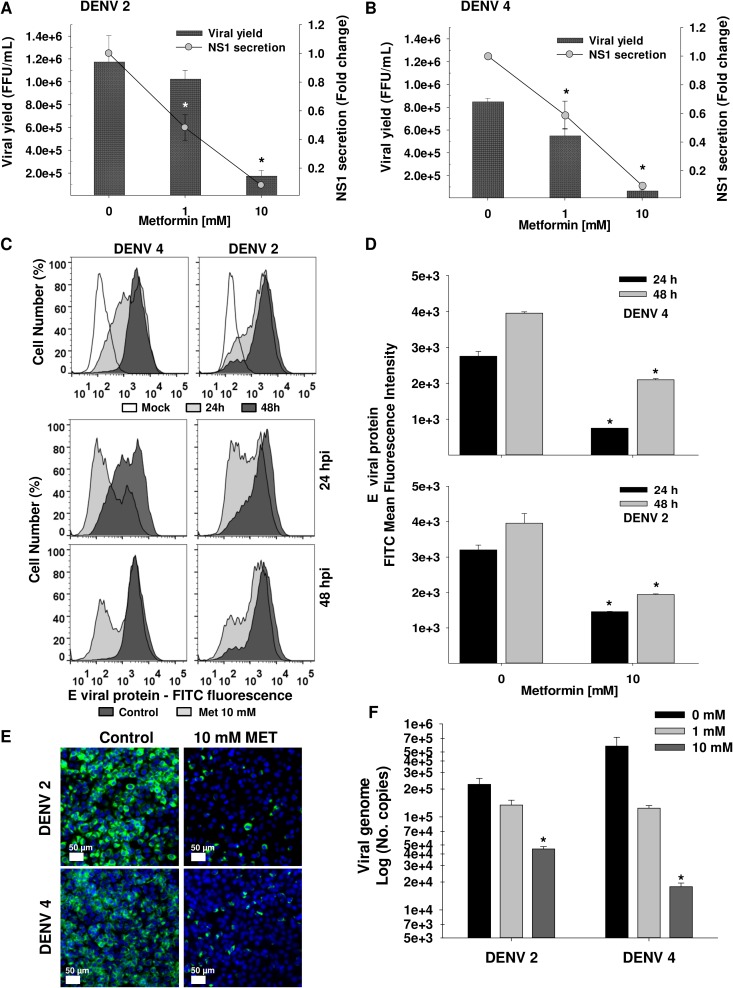
Metformin induces an antiviral effect in DENV infected cells. In **A and B**, The antiviral effect of metformin-treatment (0, 1 and 10 mM) against DENV infection was evaluated in supernatants from Huh7 cells infected (MOI 3) with DENV2 **(A)** and DENV4 **(B)** at 24 hpi through determination of viral yield by foci assay, and NS1 secretion by ELISA. Viral yield is expressed as Foci Forming Units (FFU) / mL. NS1 secretion was normalized respect to infected non-treated cells and expressed as fold change vs 0 mM. **(C)** The percentage of infected cells after 10 mM metformin-treatment was determined by flow cytometry using a mouse anti-E monoclonal antibody-4G2 to detect the E viral protein in mock or DENV 2/4 infected cells. ***Upper histograms*** show the fluorescence of infected cells at 24h *(gray filled histograms)* or 48h *(dark filled histograms)* respect to mock infected cells *(non-filled histograms)*. ***Lower histograms*** display the fluorescence of DENV infected cells treated with metformin *(gray filled histograms)* respect to vehicle-treated infected cells *(dark filled histograms)*. **(D)** The Mean Fluorescence intensity (MFI) is presented on Graphs. **(E)** DENV 2/4 infected cells treated with 10mM metformin (MET) were visualized at 24 hpi by confocal microscopy using a mouse anti-E monoclonal antibody-4G2 ***(green)***. Nuclei were stained with Hoechst ***(blue)***. Scale bar 50 μm. Images correspond to one experiment representative of *n = 3*. **(F)** The number of viral genome copies of DENV 2/4 infected cells treated with metformin (0, 1, 10 mM) for 24h was examined by qRT-PCR, and expressed as Log of No. Copies. DMSO 0.5% was used as vehicle for all cases (0 mM). Data are means ± S.E of *n = 3* independent experiments realized by duplicated. * p<0.*05* compared to non-treated cells.

The replicative complexes disruption promoted by the activation of AMPK supports our hypothesis that this compound could inflict important inhibition of DENV infection. To further analysis of the antiviral effect induced by the activation of AMPK, DENV2 infected Huh7 cells were treated for 24 h with increasing concentrations of direct and indirect AMPK activators (MET, TMPA and A-769662) and lovastatin and infection was evaluated by percentage of infected cells (FACS) and viral yield (foci assay). The reduction in amount of infected cells promoted by all the drugs was concentration-dependent, The discrepancies between drugs could be explained by the concentrations used of each drug (**S5A and S5B Fig**). Cell viability assay in the presence of the different drugs is presented in supplemental material (**S7 Fig**). Additionally, viral yield was reduced by AMPK activation and lovastatin. Particularly, A769662 reduced viral yield for 2 log meanwhile other drugs reduced viral progeny for 1 log, however this drug just reduced the amount of infected cells just in 15% suggesting that the analog of AMP has an important effect in viral particle assembly or secretion (**S5C and S5D Fig**). From the AMPK activators tested in this work, the only drug approved by FDA is MET, then, we considered to evaluate the antiviral effect of this drug in a deeper way. It was confirmed that MET-treatment declined in a dose-dependent manner the viral yield up to one logarithm and the NS1 secretion up to 90% in infected cells with both serotype DENV2 (**Fig 6A**) and DENV4 (**Fig 6B**) at 24 hpi. In addition, the inhibition of DENV infection was demonstrated by flow cytometry, confocal microscopy and viral genome amplification (qRT-PCR). The 10 mM MET-treatment decreased the amount of infected cells with both serotype DENV2 and DENV4 at 24 and 48 hpi compared to non-treated cells (**Fig 6C lower panels darker histograms and 6D**). Images by confocal microscopy showed a significant reduction in DENV2 and DENV4 infected cells (**Fig 6E, green color cells**) after 10 mM metformin-treatment at 24 hpi compared to non-treated cells. Finally, a dose-dependent reduction in viral genome copies (up to 0.7 logarithm for DENV2, and 1.5 logarithm for DENV4) was observed in infected cells after metformin-treatment at 24 hpi respect to non-treated cells (**Fig 6F**). These data demonstrate that MET has a significant anti-DENV effect without affects the cell viability **(S7 Fig).**

Further evidence supporting AMPK importance on DENV replicative cycle was obtained using non-cytotoxic concentration of compound C (CC), an AMPK inhibitor (**S7, S8A and S8B Figs**). DENV infected cells (0.3 MOI) treated with CC for 24 h (clear histograms) showed a greater viral infection than non-treated cells (dark histograms), which was depicted as the rightward shift of clear histograms (DENV CC-treated cells) respect to dark histograms (DENV non-treated cells) (**S8A Fig**), and quantified as the mean fluorescence intensity (**S8B Fig**). AMPK inhibition (CC-treatment) raised the viral genome copies up to a half logarithm in DENV 2 and up to 0.7 logarithm in DENV 4 infected cells supporting that AMPK modulation is a key step in the DENV infection (**S8C Fig**). Together, our results highlights AMPK as an important host cell factor required during DENV infection, which could be a new therapeutic target against viral infection by DENV.

### DENV does not alter PP2A phosphatase activity but its inhibition has an antiviral effect

**Fig 7:**
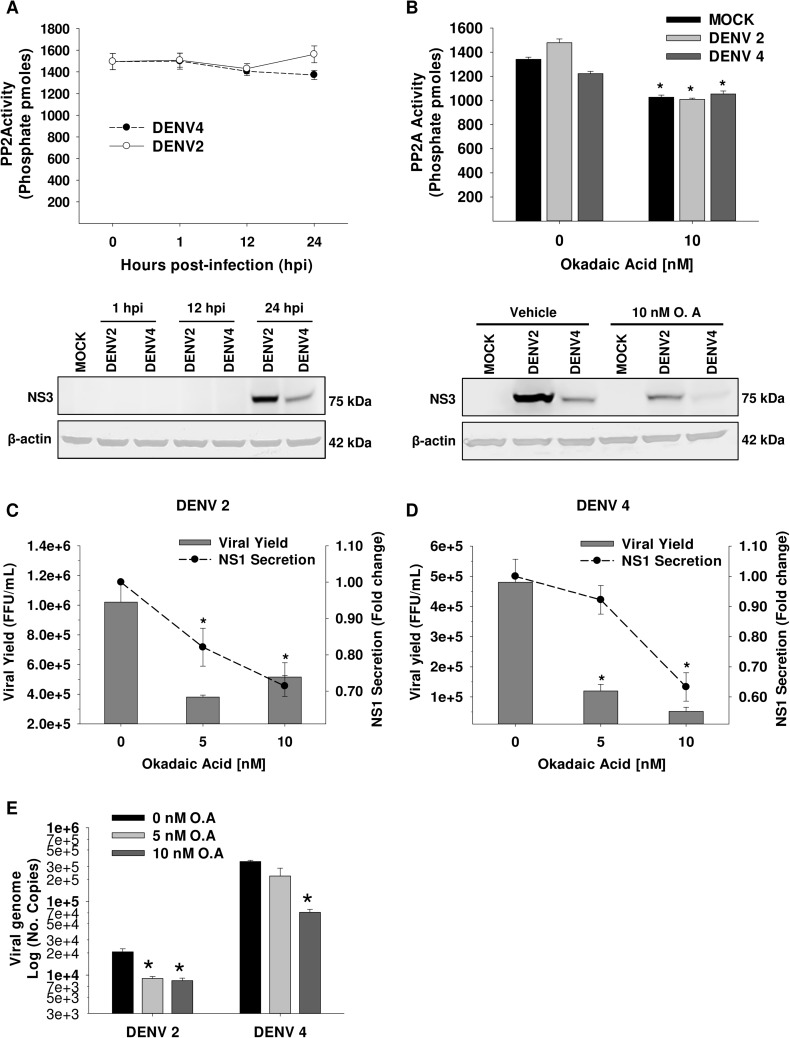
PP2A activity is not altered by DENV, but its inhibition by Okadaic acid has an antiviral effect. The PP2A activity was analyzed in Huh7 cells infected (MOI 3) with DENV 2/4 at 1, 12 and 24 hpi **(A)**, and in Mock or DENV 2/4 infected cells treated with DMSO 0.05% (vehicle) or 10 nM Okadaic acid (O. A) for 24h **(B)**. Activity is expressed as picomoles of phosphate (phosphates pmoles). From the same cell lysates, the levels of NS3 viral protein *(lower panels)* were determined by WB as infection test. * p<0.*05* compared to mock vehicle-treated cells. The antiviral effect of O. A (0, 1 and 10 nM) against DENV infection was evaluated in supernatants from Huh7 cells infected (MOI 3) with DENV2 **(C)** and DENV4 **(D)** by viral yield and NS1 secretion at 24 hpi. Viral yield is expressed as Foci Forming Units (FFU) / mL. NS1 secretion was normalized respect to infected non-treated cells and expressed as fold change vs 0 mM. **(E)** The number of viral genome copies of DENV 2/4 infected cells treated with O. A (0, 5, 10 nM) for 24h was examined by qRT-PCR, and expressed as Log of No. Copies. * p<0.*05* compared to non-treated cells. DMSO 0.05% was used as vehicle for all cases (0 nM). Data are means ± S.E of n = 3 independent experiments realized by duplicated.

PP2A dephosphorylates HMGCR and boosts its activity [34,35]. Previously in this work we showed that PP2A inhibited by OA does not alter the HMGCR activity up-regulation during DENV infection, suggesting that DENV infection does not alter the phosphatase activity of PP2A. To rule out that PP2A was also modulated in response to DENV infection, its phosphatase activity was evaluated in DENV 2/4 infected cells at 1, 12 and 24 hpi. DENV infection did not induce any effect on PP2A phosphatase activity (**Fig 7A**). This was corroborated in mock and DENV infected cells in the presence and absence of okadaic acid for 24h. As expected, OA-treatment diminished the PP2A phosphatase activity compared to non-treated cells but not respect to mock infected cells, which demonstrated that DENV infection does not alter PP2A phosphatase activity (**Fig 7B**).

Interestingly, OA-treatment with non-cytotoxic concentrations **(S7 Fig)** lowered the viral protein levels in DENV 2/4 infected cells (**Figs 7B lower panel and 2C**), it decayed the viral yield up to a half logarithm as well as the NS1 secretion up to 30% for both DENV serotypes (**Fig 7C and 7D**), and it diminished viral genome copies (**Fig 7E**) compared to DENV non-treated cells. These data suggest that modulation of PP2A phosphatase activity by okadaic acid elicits an anti-DENV effect.

## Discussion

It has been suggested that DENV modulates the cellular lipid metabolism in order to boost *de novo* synthesis of membranous systems (replicative complexes) for viral replication [22,30,49,50]. The antiviral properties of drugs that disrupt fatty acid and cholesterol biosynthesis pathways against DENV suggest a pivotal role for lipidic content during the infection process [26–28]. Previously, our group reported high intracellular cholesterol levels at early times after DENV infection in parallel to an enhanced activity of the cholesterol-limiting enzyme HMGCR, evidenced as a reduction of phosphorylation levels [33]. However, molecular mechanisms involved in the enzyme activation have not been studied in detail. In this work, we demonstrated that the raised HMGCR activity prompted by DENV infection was mediated by a reduction in the AMPK kinase activity (phosphorylation at Thr-172), which avoid the HMGCR phosphorylation and results in an increased HMGCR activity. The HMGCR activity increased by DENV was not modulated by PP2A, an activator phosphatase of HMGCR. The rise in HMGCR activity stimulated a cholesterol accumulation on endoplasmic reticulum (ER) needed for DENV-replicative complexes formation and maintenance of its architecture, as was evidenced by treatment with MET- (AMPK activator) and lovastatin (HMGCR inhibitor). Although lovastatin and MET induce a reduction in HMGCR activity, lovastatin acts only on the activity of HMGCR, while metformin is increasing the activity of AMPK, one of the most important host metabolism regulator[51] impacting also in the fatty acid synthesis which has been reported as important lipids for DENV infection [51]. In concordance, AMPK activation by TMPA and A769662-treatment showed a significant antiviral effect. On the other hand, DENV did not alter the PP2A activity but its inhibition by OA prompted an HMGCR-independent antiviral effect. These results contribute to explain the mechanism involved in the modification of host lipid metabolism during DENV replicative cycle, and identify new potential antiviral targets for DENV replication.

AMPK is a master metabolic controller that could be up- or down-modulated by different viruses to provide a favorable host lipidic environment for its replication. The modulation of AMPK has been studied during the infection with other viruses such as hepatitis C virus (HCV), where it was observed the inhibition of this kinase and the concomitant intracellular lipids accumulation [52–54]. As well, the latent membrane protein 1 from Epstein-Bar virus (EBV) inhibits the AMPK activity and the subsequent downstream effectors activation [55]. Similar to previous reports, we observed that DENV infection downregulates AMPK activity at early times post-infection. AMPK activity is controlled via direct dephosphorylation by PP2C [56], and the allosteric action of AKT, which when its activated phosphorylates AMPK α2-subunits at ser-485/ser-491 reducing the Thr-172 phosphorylation and therefore the AMPK activity [57]. In fact, an elevated AKT activity in cells infected with Japanese encephalitis virus (JEV) and DENV has been observed [58]. Hence, the alteration of upstream effectors such as PP2C and AKT could be one of the mechanisms through which DENV reduces the AMPK phosphorylation at Thr-172 (kinase activity).

An opposite effect was observed during Human Cytomegalovirus (HCMV) infection, where AMPK activation was augmented in order to trigger the glucose import and the glycolytic pathway essential during HCMV replication [59]. The translocation of the Liver Kinase B1 (LKB1) from the nucleus to the cytoplasm and its subsequent phosphorylation were found to be the crucial for activation of AMPK [47,60]. In turn, the MEK1/2 [mitogen-activated protein kinase (MAPK) extracellular signal-regulated kinase (ERK) kinase]/ERK1/2 signaling pathway prevents the LKB1-AMPK signaling [61], thus the stimulation of the MEK/ERK pathway in DENV infected cells could explain the depletion of AMPK phosphorylation. Several authors demonstrated that DENV [62] and others flaviviruses including Hepatitis C Virus (HCV) [63], West Nile fever virus (WNV) [64], Japanese encephalitis virus (JEV) [65] and yellow fever virus (YFV) [66] cause the turn on of the MEK/ERK signaling.

Furthermore, AMPK activity may be modulated by viral factors. NS4A is an important replicative complex component, it has four transmembrane domains (pTMSs1-4), pTMS4, known as 2k fragment, is cleaved from the mature NS4A and the expression of cleaved NS4A is enough to induce the membrane alterations observed in DENV infected cells indicating a crucial role of NS4A in the replicative complex formation [41,42]. Thus, it is feasible that NS4A could modulate the AMPK activity, which is our current focus. NS1 has been observed attached to lipid rafts, suggesting that NS1 may incite the signaling associated with these membrane regions such as the PI3K/AKT pathway, which in turns inactivates AMPK [67]. Then, expression of individual viral proteins could help to study whether there are viral factors implicated in the AMPK activity down-modulation in DENV infected cells.

DENV infection prompts a membrane rearrangement in order to build up the replicative complexes, which necessitates the modification of the host lipid metabolism. It has been reported that during DENV infection the Fatty Acid Synthase (FAS) is redistributed to replicative complexes through interaction between NS3 viral protein and Rab18, a small GTPase involved in the regulation of membrane trafficking and reorganization [68], likely to supply the free fatty acids needed for the membrane remodeling crucial for virus replication [29,30]. In the same way, our findings showed that HMGCR co-localized with NS4A and NS3, viral components involved in viral replication and localized at replicative complexes. This co-localization was disrupted by AMPK activation with metformin or HMGCR inhibition with lovastatin, suggesting that replicative complexes formation demands an activated HMGCR. Moreover, cholesterol was accumulated at replicative complexes. An important question to answer was if the absence of cholesterol inhibited the RC formation or if the low amount of non-structural proteins produced during treatment with the drugs caused a reduction in the RC formation. To analyze these two possibilities, cells were transfected with NS4A-eGFP that showed a compact distribution in the perinuclear region such as the RC. Treatments with AMPK activators and lovastatin induced a disperse distribution of NS4A, supporting the idea that the absence of cholesterol induced by drugs inhibited the ability of NS4A to be distributed to the perinuclear region and to form RC.

AMPK participates in the maintaining of cellular lipid homeostasis through inhibition of limiting-enzymes of lipid anabolic pathways such as HMGCR, involved in cholesterol synthesis; ACC, involved in the fatty acid synthesis; FAS, involved on free fatty acid anabolism; among others [37,69]. The ACC is one of the best characterized downstream targets of AMPK, the inhibition of ACC by AMPK together with the FAS inhibition (recruited to replicative complexes) has a strong impact on the biosynthesis of fatty acids. Besides, AMPK interacts with the SREBP-2 (sterol regulatory element binding protein 2) triggering its phosphorylation, which prevents its nuclear translocation reducing the HMGCR transcription [37,70,71]. Hence, our results suggest that AMPK activity inhibition could be an important mechanism through lipid metabolism is regulated during DENV in order to boost cholesterol and fatty acids synthesis, which is required for ER membrane reorganization necessary to build and maintain replicative complexes. AMPK activation inhibits several lipogenic pathways [72], this could explain the higher reduction of viral protein levels induced by MET with respect to lovastatin treatment.

The key role of AMPK during DENV infection observed in the current work was supported by the significant antiviral effect occasioned for metformin treatment, a pharmacological activator of AMPK. Such effect is consistent with the antiviral effect of NDGA (Nordihydroguaiaretic acid) [32], a compound derived from *Larrea tridentata* that also is able to over activates AMPK [73,74]. NDGA inhibits efficiently viral replication and morphogenesis [32]. The antiviral effect inflicted by the pharmacological activation of AMPK using other different AMPK activators (AICAR and A769662) has also been reported for other classes of viruses such as Rift Valley Fever Virus (RVFV; *Bunyaviridae*: *Phlebovirus*), Hepatitis C virus (HCV; *Flaviviridae*: *hepacivirus*) [52,75,76], Sindbis (SINV; *Togaviridae*: *alphavirus*), West Nile Virus (WNV; *Flaviviridae*: *flavivirus*) and Vesicular Stomatitis Virus (VSV; *Rhabdoviridae*: *vesiculovirus*), which suggest that AMPK and the inhibition of lipid biosynthesis could have a role in the intrinsic innate immune response [37]. In fact, several viruses inhibit the AMPK pathway to allow the viral replication [77], as we observed in the current work during DENV infection.

A recent study reveals that limiting flux through the cholesterol biosynthetic pathway spontaneously engages a type I IFN response in a STING-dependent manner [78]. More recently, it has been described that AMPK promotes innate immunity and antiviral defense through modulation of Stimulator of Interferon Genes (STING) Signaling [79]. It is well known that STING is a crucial adaptor in immune cells after infection with DENV and it is the target for cleavage and degradation by the viral protein NS2B-NS3 to inhibit innate immune responses [80]. Therefore, mevalonate pathway and IFN-signaling pathway are part of a metabolic-inflammatory circuit that ensures any changes in the activity of one pathway are sensed by the other pathway. DENV infection triggers a robust IFNα/β response. However, DENV nonstructural proteins are capable of a down-regulation of the JAK/STAT pathway, inhibiting the gene expression directed by interferon [80–85]. In consequence, it is important to analyze the relevance of cholesterol modulation and AMPK activity in IFN type I synthesis during DENV infection.

On the other hand, HMGCR activity could be also modulated by the protein phosphatase 2A (PP2A). Here, we observed that DENV infection does not affect the PP2A activity and we demonstrated that the raised HMGCR activity during infection was non-dependent of PP2A. Interestingly, the inhibition of PP2A activity by OA showed an antiviral effect in an HMGCR-independent manner. During HCV infection it has been observed that overexpression of PP2A drives to a greater NS3 helicase activity and inhibits the signaling by interferon through the inhibition of STAT1 and STAT2 pathways [86–88]. Therefore, it is possible that OA could amplify the interferon response inhibiting the DENV infection. Alternatively, it is interesting analyze the relation between the activity of NS3 helicase and PP2A in order to elucidate whether the antiviral effect is mediated by the direct inhibition of NS3 activity, or the interferon response activation, or both of them. Additionally, OA activates the protein kinase depending of RNA (PKR) that has a well-known and established antiviral effect [89,90]. Thus, the determination of NS3 helicase activity and PKR activity in DENV infected cells could be useful to characterize the antiviral mechanism caused by OA.

Overall, our data demonstrated that DENV modulates negatively the AMPK activity promoting an increase of HMGCR-reductase activity that lead to a cholesterol accumulation in the replication sites, an effect necessary to maintain the replication complexes architecture. This work supports the cholesterol role on building and maintenance of DENV replicative complexes, and suggests that AMPK modulation is the main pathway through which DENV modifies the host lipid metabolism during its replicative cycle. The significant dose-dependent antiviral effect of MET and the other AMPK activators observed in this work highlights AMPK as a new potential antiviral target, and suggests that metformin, a well-known and widely used hypoglycemiant drug, may be a good candidate against DENV. It has been reported that AMPK activation is required for MET inhibitory effect on glucose production, thus, the concentration of MET used in diabetes mellitus patients should induce a reduction in the HMGCR activity. Under this perspective, it could be suggested that the use of therapeutically doses of MET in healthy patient could increase AMPK activity, inhibiting the HMGCR function generating a beneficial effects during DENV infection. This suggesting can not be extrapolated to type 2 diabetes patients treated with MET because present evidence indicates that diabetes *per se*, adversely influence the clinical presentation of any infection because patients have dysfunction in several organs and systems [91] and impairment in host defenses mechanisms [92]. Specifically, type 2 diabetes patients have endothelial dysfunction, which can condition more severe forms of dengue infection[93]. In this aspect, there are no specific studies, which determine dengue severity in diabetic patients treated or not with metformin. The only clinical trial reported with dengue patients is the one presented by Whitehorn et al. They performed a randomized, blind, placebo controlled trial of 5 days of 80 mg of lovastatin vs placebo in 300 Vietnamese adults. They found no evidence of a beneficial effect on any of the clinical manifestation or on dengue viremia [94]. However the study has some limitations. First, severe dengue occurred infrequently in the study population (1%) thus, a bigger sample is needed to conclude about the influence of the drug in severe forms of dengue infection. Second, they administered the drug within 72 hours of illness onset, it is possible that earlier intervention could be necessary to improve outcome in severe cases. Further analysis in animal models and phase 3b clinical trials are essential to determine if the use of MET or other AMPK activators as an anti-dengue treatment is feasible.

## Materials and methods

### Cell culture, viral strains and drugs

The differentiated hepatocyte derived cellular carcinoma cell line Huh7 (kindly donated by Dr. Rivas from Universidad Autonoma de Nuevo Leon) and Green monkey kidney cells (Vero) (ATCC) were cultured in advanced DMEM supplemented with 2 mM glutamine, penicillin (5x10^4^U/ml)-streptomycin (50μg/ml), 5% fetal calf serum (FCS), and 1 ml/l of amphotericin B (Fungizone) at 37°C in a 5% CO_2_ humidified atmosphere. The DENV propagation, serotype 4 H241 strain and serotype 2 New Guinea strain, was carried out in CD1 suckling mice brains (provided by Unidad de Producción y Experimentación de Animales de Laboratorio (UPEAL)). The titers were determined by plaque assays in BHK-21 cells (kindly donated by Instituto Pedro Kouri, Cuba) as was previously described [95]. CD1 suckling mice brains from mock-infected mice were used as control. AMPK activators Metformin and A769662 were obtained from Abcam Biochemicals (catalog number ab120847 and ab120335 respectively), TMPA (492910) and Compound C (171261) from EMD Millipore, lovastatin from Sigma (M2147) and okadaic acid from Cell signaling (#5934).

### DENV infection and treatment

The Huh7 cells were seeded in an culturing-format appropriate, and when they reached a 70–80% of confluence were washed and infected with DENV 2/4 (serotype 2 or 4) at a multiplicity of infection (MOI) of 3 (0.3 for compound C-treatment) in medium with 1% serum for 2h at 37°C. After infection, cells were washed once with glycine (pH 3) to inactivate non-internalized virus and 3 times with PBS, and posteriori they were treated with the indicated treatment (metformin, Compound C, okadaic acid or lovastatin) in complete medium for 24h or 48h at 37°C. All experiments were conducted following the same infection protocol and drug-treatment scheme.

### Determination of AMPK phosphorylation by ELISA

The measurement of AMPK phosphorylation levels was evaluated by enzyme-linked immunosorbent assay (ELISA) using the kit AMPKα [*p*T172] (Invitrogen) following the manufacturer’ instructions. Briefly, 1.2x10^6^ cells were platted in 60 mm dishes. After 24h, cells were mock or DENV infected, and were treated with vehicle (DMSO 0.5%), metformin 10 mM (AMPK activator) or compound C 10 μM (AMPK inhibitor). Cells were lysed in buffer containing 10 mM Tris, pH 7.4, 100 mM NaCl, 1 mM EDTA, 1mM EGTA, 1 mM NaF, 20 mM Na_4_P_2_O_7_, 2 mM Na_3_VO_4_, 1% Triton X-100, 10% glycerol, 0.1% SDS, 0.5% deoxycholate, 1 mM PMSF, and protease inhibitor Complete Mini, EDTA-free (ROCHE). The standard curve was performed following the manufacturer’s instructions and 100 μg of protein for sample were analyzed interpolating each sample value in the curve. The phosphorylation levels of AMPK were expressed as U/mL.

### HMGCR assay activity

The enzymatic activity of HMGCR was evaluated by quantitation of the NADPH extinction using the HMG-CoA reductase assay kit (SIGMA-ALDRICH). Since the reduction of HMG-CoA to mevalonate and CoA-SH catalyzed by HMGCR implicates the oxidation of NADPH to NADP^+^, the reduction in the amount of NADPH indicates the HMGCR activity, which can be quantified by spectrometry. Briefly, 1.2x10^6^ Huh7 cells were platted in 60 mm dishes, after 24h cells were mock or DENV infected, and were treated with DMSO 0.5% (Vehicle), metformin (AMPK activator), okadaic acid (PP2A inhibitor) and lovastatin (HMGCR inhibitor). After 24 hpi, cells were lysated, and 10 μL from clarified cell lysate were analyzed in a microplate reader (Tekan) at λ = 340 nm every 20 seconds during 20 minutes at 37°C. A recombinant HGMCR provided by the kit was used as a positive control, 10 μL lysis buffer were used as blank. The activity is expressed as U/mg protein where 1 unit (U) is the amount of HMGCR oxidating 1μmol of NADPH in a minute.

### PP2A enzymatic assay

The PP2A activity was evaluated by the PP2A immunoprecipitation phosphatase assay kit (Merck-Millipore). Cell lysates were obtained from mock or DENV infected cells treated with DMSO 0.05% (0 nM) or okadaic acid (10 nM), and 100 μg of protein were analyzed following the manufacturer recommendations. Briefly, the PP2A immunoprecipitation was performed using a specific antibody coupled to agarose beads, the beads were incubated with a specific threonin phospho-peptide (K-R-pT-I-R-R), and the PP2A catalytic activity was analyzed using an ELx808 BioTek plate reader. The PP2A activity is indicated by the phosphate released and its reaction with malachite green. The values obtained for each sample were interpolated in a phosphates standard curve. The PP2A activity was expressed as picomoles (pmol) of phosphate.

### Western blot analysis

The same cell lysates used for enzyme activity determinations and AMPK phosphorylation were analyzed by western blot (WB) in order to determine the presence of the NS3 protein as infection control. Fifty micrograms of protein were assayed by SDS-PAGE and immunoblotting using a NS3 rabbit polyclonal antibody (GENETEX), and an actin mouse monoclonal antibody as load control. For additional determinations of viral proteins rabbit anti E protein (GENETEX) and rabbit anti prM protein (GENETEX) were used. To determine the levels of *p*-AMPKα and total AMPKα rabbit monoclonal *p*-AMPKα [T172] and mouse AMPKα monoclonal antibody (both from Cell Signaling) were used.

### Confocal microscopy and flow cytometry (FACS)

Huh7 cells infected and treated as indicated before were analyzed by confocal microscopy and flow cytometry to determine the viral proteins distribution, infected cells percentage and HMGCR levels. The slides or harvested cells were fixed with 1% formaldehyde, permeabilized for 20 min (1X PBS, 0.1% saponin and 1% FBS), and incubated for 2 h at room temperature (RT) with rabbit anti-NS3 polyclonal antibody (GENETEX), rabbit anti-NS4A polyclonal antibody (GENETEX) or mouse anti-E monoclonal antibody (4G2) to stain the viral proteins, double strand RNA antibody (MAB J2 anti-dsRNA kindly donated by Garcia-Blanco/Bradrick lab from University of Texas Medical Branch), and goat anti-HMGCR (Santa Cruz Biotechnologies) to stain the cellular enzyme. As secondary antibodies were used donkey anti-mouse Alexa 488, donkey anti-goat Alexa 555, donkey anti-rabbit Alexa 647 or chicken anti-rabbit Alexa 488 (Life technologies). Intracellular cholesterol was stained with the fluorescent dye filipin III complex (Sigma) and nuclei were counterstained with Hoechst 33342 or propidium iodide (Life Technologies). Slides were observed in a Zeiss LSM700 laser confocal microscopy and images were analyzed using the ZEN software, v. 2010. The same software was used to determine colocalization, the amount of infected cells analyzed for colocalization ratios between viral proteins, HMGCR and cholesterol. Flow cytometry was performed in a BD LSR Fortessa and data were analyzed with the FlowJo v. 10 software.

### Electron microscopy

Huh7 infected cells were harvested at 24 hpi, pelleted, and fixed with 4% paraformaldehyde and 0.5% glutaraldehyde in PBS for 1h at RT. Then, the pellet was gradually dehydrated with growing concentrations of ethanol (25, 50, 70, 90 and 100%). Samples were embedded in LR-white resin and polymerized with UV light at 4°C for 48 h. Slides were processed in an Ultracut E microtome (Reichter-Jung) and mounted in nickel grids. The NS4A and HMGCR were detected using a rabbit anti-NS4A antibody (Genetex) and goat anti-HMGCR antibody (Santa Cruz Biotechnology) 1:10 dilution. Anti-rabbit (Ted Pella Inc) and anti-goat (Electron Microscopy Science) coupled to gold particle were used to detect the proteins of interest. Samples were analyzed a Transmission Electronic Microscope Jeol JEM-1011.

### Viral yield and NS1 secretion

Supernatants from infected and treated cells were analyzed to determine the viral yield by Foci Forming Units assay. Confluent monolayers of Vero cells grown in 96-well plates were inoculated with serial dilutions of supernatant media from DENV-infected cells (final volume 50 μL) for 1 h at 37°C to allow the viral absorption. Then, the inoculum was removed, 0.2 mL of complete media were added and cell monolayers were washed once with Hank's solution. The medium was removed at 24 hpi and cells were fixed with 1% formaldehyde, permeabilized for 20 min, incubated with anti-E 4G2 monoclonal antibody for 2h at RT, and detected with anti-mouse-FITC secondary antibody. Foci were determined by fluorescence microscopy and expressed as Foci Forming Units (FFU) / mL. NS1 secretion was measured in supernatants by ELISA (Platelia, Biorad) [96].

### Evaluation of viral genome by qRT-PCR

To amplified the interest fragment of the viral genome, which correspond to a 151-pb fragment of the DENV capsid gene, a conventional RT-PCR of RNA isolated from DENV-infected cells was performed using the following primers: DV2C-L 5´-CAA TAT GCT GAA ACG CGA GA-3´and DV2C-R: 5´-TGC TGT TGG TGG GAT TGT TA-3´. This product was cloned in a pJet1.2 Vector System (Thermo scientific), and the recombinant plasmid was purified and quantified by spectrophotometry at λ = 260 nm. A dilution containing 10^10^ copies of plasmid/mL was prepared according to the formula:

$$No.\;Copies=\frac{6\times 10^{23}\;copies/mol\times concentration\;[g/µl]}{plasmid\;molecular\;weight}+insert\;[g/µl]$$

Serial dilutions of the plasmid (10^9^–10^2^ copies/ml) were prepared and a standard curve was generated.

The cDNA was obtained by reverse transcription using 1 μg of total RNA from each experimental condition, random primers (0.025 μg/μl each) (Promega), and the enzyme ImpromII (Promega) during 1 cycle of amplification under the following conditions: 25°C for 5 min, 42°C for 60 min, and 70°C for 15 min (Veriti Thermal Cycler, Applied Biosystems). The real-time PCR amplification was made by SYBR Fast universal (Kapa) in an Eco Illumina System apparatus using a reaction mix containing 1 μl of cDNA and 5 μl of 2X Master Mix under the following conditions: 2 min at 50°C, 2 min at 95°C, 40 cycles of 5 sec at 95°C, and 30 sec at 55°C. To confirm primer dimer absence, a dissociation curve was made by heating of products since 55°C to 95°C.

### Cell viability assay

Huh7 cells (30,000) were platted in 96 well/plate. After 24 hours the cells were treated with vehicle (DMSO 0.5%), metformin (MET), TMPA, A769662, compound C (CC), Lovastatin (LOV) and Okadaic Acid at concentrations mentioned in the supplemental Fig 6 during 24 hours. Later, cell viability was assayed using cell titer 96 Aqueous one solution Reagent (Promega) following the manufacturer´s protocol.

### Statistical analysis

Differences between treatments and control groups were evaluated using the SigmaPlot/Stat package 11. In all cases, parametric or nonparametric tests and the appropriate post-hoc test were applied. If data met with the analysis of variance (ANOVA) assumptions of normality and equal variance (parametric), a one-way ANOVA was done followed by a Holm-Sidak multiple comparison post-hoc test. Data did not met with ANOVA assumptions (nonparametric) were analyzed using a Kruskal–Wallis one-way ANOVA on ranks followed by Dunnett multiple comparisons post-hoc test. For some cases, a *t*-test (parametric) or Mann–Whitney U test (nonparametric) was conducted. A *p<0*.*05* was considered as statistically significant. Data were plotted as mean ± standard error of the mean (SEM).

### Ethics statement

This study was conducted in accordance with the Official Mexican Standard Guidelines for Production, Care and Use of Laboratory Animals (NOM-062-ZOO-1999) and the protocol, number 048–02, was approved by the Animal Care and Use Committee (CICUAL) at CINVESTAV-IPN, Mexico.

## Supporting information

S1 Fig Intracellular distribution of HMGCR and NS4A viral protein during DENV infection using electron microscopy. The closeness between both proteins was corroborated by electron microscopy in DENV4 infected Huh7 cells using a goat anti-HMGCR antibody coupled to a 10 nm gold particle **(Small dots, HMGCR)**, and a rabbit anti-NS4A antibody coupled to a 30 nm gold particle **(Big dots, NS4A viral protein)**. Scale bar 500 nm. Broken squares depict the area of magnification for panels indicated as ZOOM. Images correspond to one experiment representative of n = 2 independent experiments realized by duplicate. ER: endoplasmic reticulum.
(TIF)

S2 Fig HMGCR and NS3 protein distribution and colocalization. The distribution of HMGCR and components of viral replication complexes (NS3 and E viral proteins) was evaluated by confocal microscopy in Huh7 cells infected with DENV2 (MOI 3) and treated with DMSO 0.5% (vehicle), 10 mM Metformin or 50 μM lovastatin (HMGCR inhibitor) for 24h. The integrity of replication complexes is depicted as the co-localization between NS3 and E protein. In A is indicated the distribution of HMGCR (red), NS3 (light blue), and E protein (green) as well as the colocalization per infected cell of HMGCR/NS3 (B) and E/NS3 (C) represented by mean ± S.E of the colocalization of 60 analyzed infected cell per condition. D and E represent the mean fluorescence intensity analyzed by flow cytometry. Graphs represent the mean fluorescence intensity ± S.E of three independent experiments, the histograms indicate the fluorescence intensity of a representative experiment.
(TIF)

S3 Fig Distribution of NS4A-eGFP in Huh7 cells in response to treatment with AMPK activators and lovastatin. Huh7 cells were transfected with a plasmid encoding NS4A-eGFP [46] and 24 hours after transfection, cells were treated with DMSO 0.5% (VEH), 10 mM metformin (MET) 100 μM TMPA, 120 μM A769662 and 50 μM lovastatin for 24 h. Distribution of NS4A-eGFP (green) was analyzed by fluorescence microscopy, the endoplasmic reticulum (RE) was stained with Concanavalin A Alexa fluor 594 (red) and nuclei with dapi (blue).
(TIF)

S4 Fig Distribution of eGFP in Huh7 cells in response to treatment with AMPK activators and lovastatin. Huh7 cells were transfected with a plasmid codifying just eGFP [46] and 24 hours after, transfected cells were treated with DMSO 0.5% (VEH), 10 mM metformin (MET) 100 μM TMPA, 120 μM A-769662 and 50 μM lovastatin for 24 h. Distribution of eGFP (green) was analyzed by fluorescence microscopy, the endoplasmic reticulum (RE) was stained with (Concanavalin A Alexa fluor 594) (red).and Nuclei with dapi (blue).
(TIF)

S5 Fig Antiviral effect of AMPK activators and lovastatin. Huh7 cells were infected with DENV2 at a MOI 3 and treated for 24 h with metformin (MET 1 mM, 5 mM and 10 mM), TMPA (25, 50, 100 μM), A-769662 (30, 60, 120 μM) and lovastatin (LOV 12.5, 25, 50 mM). Percentage of infected cells was analyzed by flow cytometry (A) and histograms (B) depicting the mean fluorescence intensity are representatives of 3 independent experiment. Viral yield from supernatants was evaluated by foci assay and represented as log_10_FFU/mL of 3 independent experiments (C), immunofluorescences are representative of 3 experiments and (D) indicate the presence of foci (green) for each condition, nuclei (blue) were stained in order to demonstrate the monolayer integrity.
(TIF)

S6 Fig Double strand RNA (dsRNA) analysis in infected cells treated with metformin. Huh7 cells were infected with DENV2 and treated with metformin10 mM or vehicle, and 24 hpi cells were fixed and stained for double strand RNA (antibody) and analyzed by flow cytometry. Graph represents the dsRNA mean fluorescence intensity from 3 experiments, Histograms are from a representative experiment.
(TIF)

S7 Fig Huh7 cell viability after metformin, TMPA, A- 769662, compound C, lovastain and okadaic acid treatment. Cell viability was evaluated in Huh7 cells treated with drugs and concentrations indicated in the graph for 24 h using the cell proliferation assay CellTiter 96 AQ_ueous_ (Promega).
(TIF)

S8 Fig AMPK inhibition induced by compound C encourages DENV infection. In **A,** The infection increase was evaluated by FACS using a mouse anti-E monoclonal antibody-4G2 to detect the E viral protein in Huh7 cells infected with Mock or DENV 2/4 (MOI 0.3), and treated with 10μM compound C (CC) or DMSO 0.5% (vehicle) for 24h. **Upper histograms** display the fluorescence of infected cells at 24h *(gray filled histograms)* respect to mock infected cells *(dark histograms)*. **Lower histograms** show the fluorescence of DENV infected cells treated with CC *(clear histograms)* respect to vehicle-treated infected cells *(dark histograms)*. **B,** The Mean Fluorescence intensity (MFI) for E viral protein is presented on Graphs. **C,** The number of viral genome copies of DENV 2/4 infected cells treated with DMSO 0.5% (vehicle) or 10μM CC for 24h was examined by qRT-PCR, and expressed as Log of No. Copies. Data are means ± S.E of n = 3 independent experiments realized by duplicated. * p<0.*05* compared to vehicle-treated cells.
(TIF)
