# Supplementary figures and images for: DENV up-regulates the HMG-CoA reductase activity through the impairment of AMPK phosphorylation: A potential antiviral target
Source: PLoS Pathog. 2017 Apr 6;13(4):e1006257. doi: 10.1371/journal.ppat.1006257 (PMC5383345; doi:10.1371/journal.ppat.1006257)

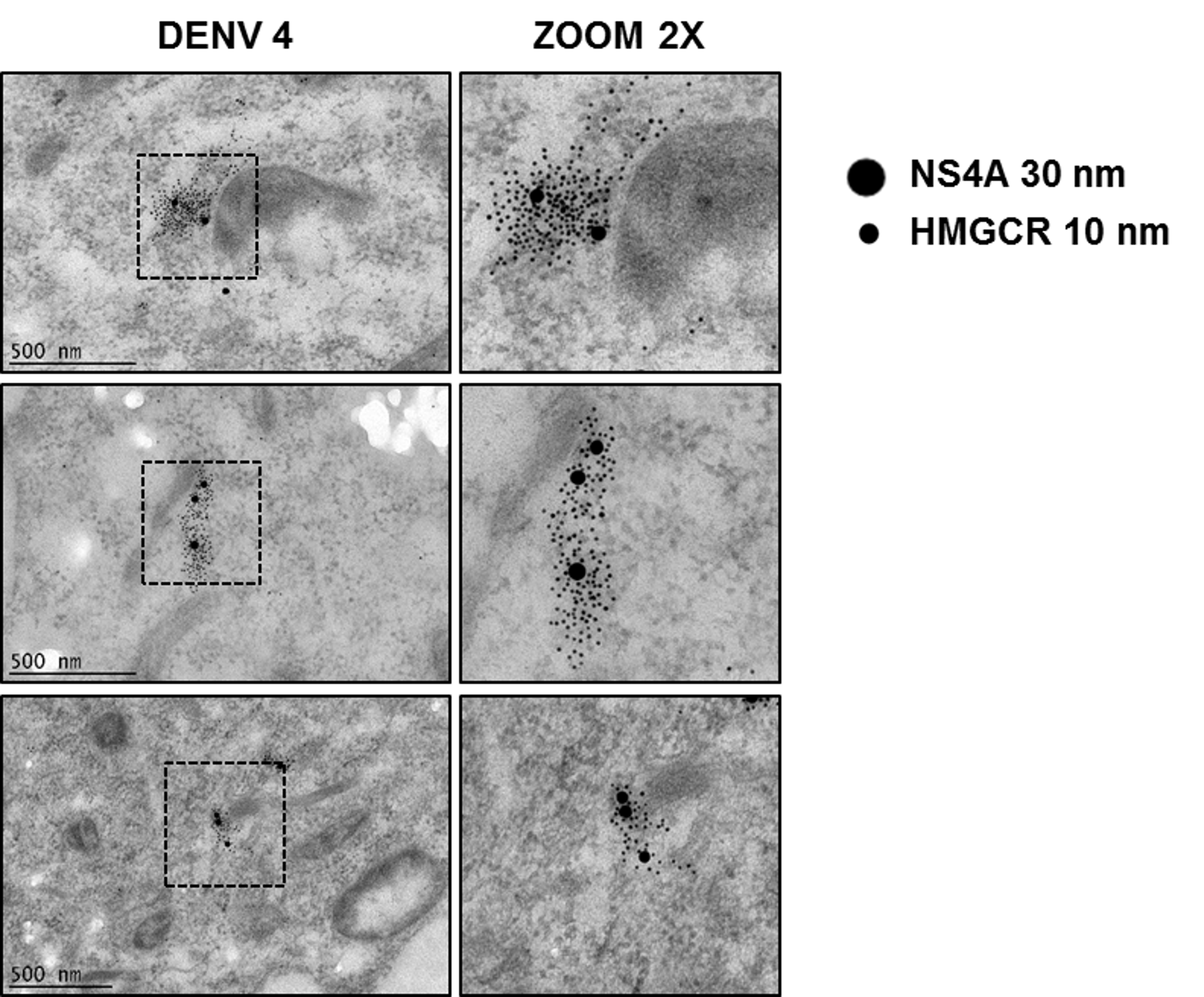

Supplement: S1 Fig — The closeness between both proteins was corroborated by electron microscopy in DENV4 infected Huh7 cells using a goat anti-HMGCR antibody coupled to a 10 nm gold particle (Small dots, HMGCR), and a rabbit anti-NS4A antibody coupled to a 30 nm gold particle (Big dots, NS4A viral protein). Scale bar 500 nm. Broken squares depict the area of magnification for panels indicated as ZOOM. Images correspond to one experiment representative of n = 2 independent experiments realized by duplicate. ER: endoplasmic reticulum. (TIF) [file ppat.1006257.s001.tif]

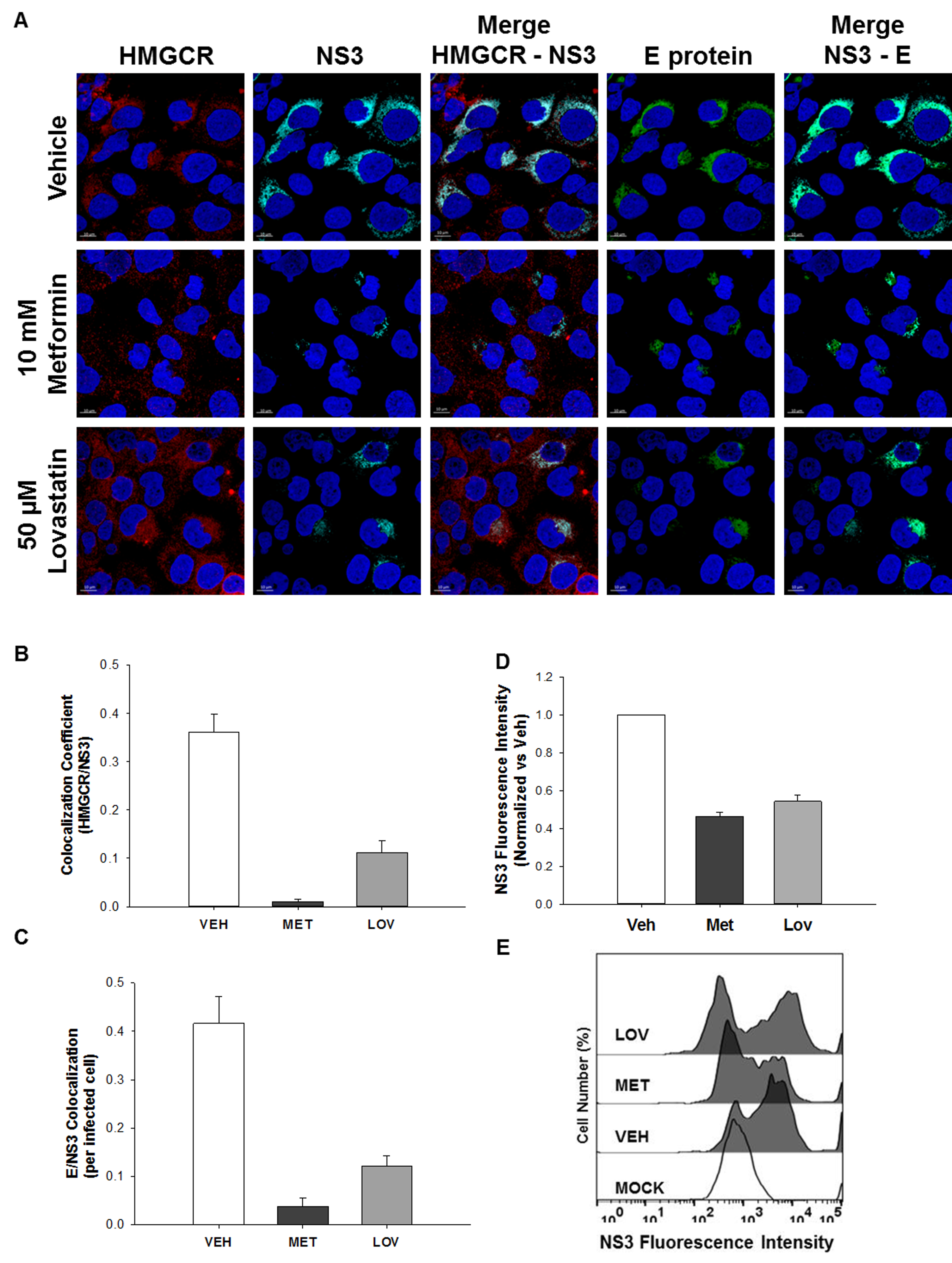

Supplement: S2 Fig — The distribution of HMGCR and components of viral replication complexes (NS3 and E viral proteins) was evaluated by confocal microscopy in Huh7 cells infected with DENV2 (MOI 3) and treated with DMSO 0.5% (vehicle), 10 mM Metformin or 50 μM lovastatin (HMGCR inhibitor) for 24h. The integrity of replication complexes is depicted as the co-localization between NS3 and E protein. In A is indicated the distribution of HMGCR (red), NS3 (light blue), and E protein (green) as well as the colocalization per infected cell of HMGCR/NS3 (B) and E/NS3 (C) represented by mean ± S.E of the colocalization of 60 analyzed infected cell per condition. D and E represent the mean fluorescence intensity analyzed by flow cytometry. Graphs represent the mean fluorescence intensity ± S.E of three independent experiments, the histograms indicate the fluorescence intensity of a representative experiment. (TIF) [file ppat.1006257.s002.tif]

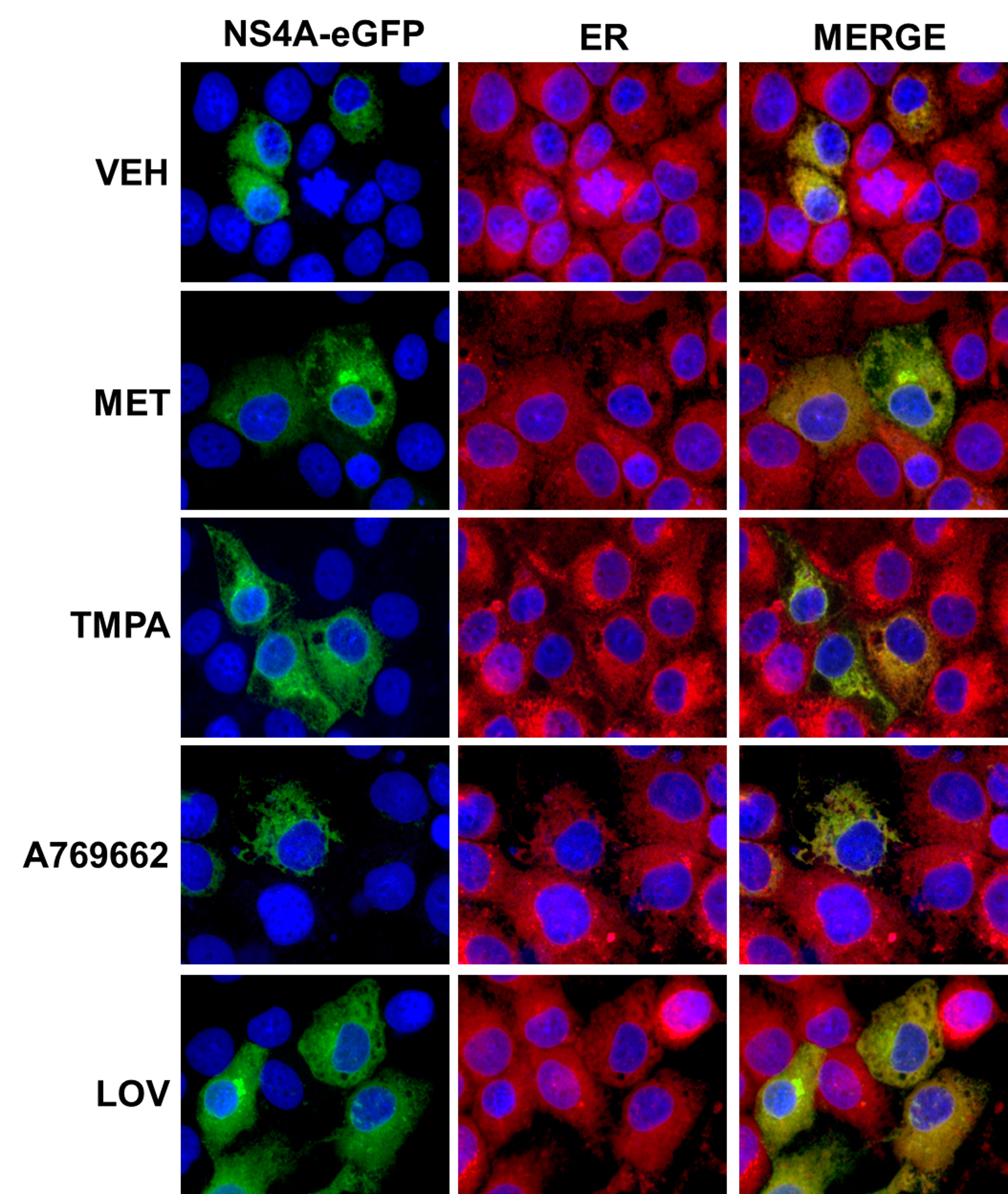

Supplement: S3 Fig — Huh7 cells were transfected with a plasmid encoding NS4A-eGFP [46] and 24 hours after transfection, cells were treated with DMSO 0.5% (VEH), 10 mM metformin (MET) 100 μM TMPA, 120 μM A769662 and 50 μM lovastatin for 24 h. Distribution of NS4A-eGFP (green) was analyzed by fluorescence microscopy, the endoplasmic reticulum (RE) was stained with Concanavalin A Alexa fluor 594 (red) and nuclei with dapi (blue). (TIF) [file ppat.1006257.s003.tif]

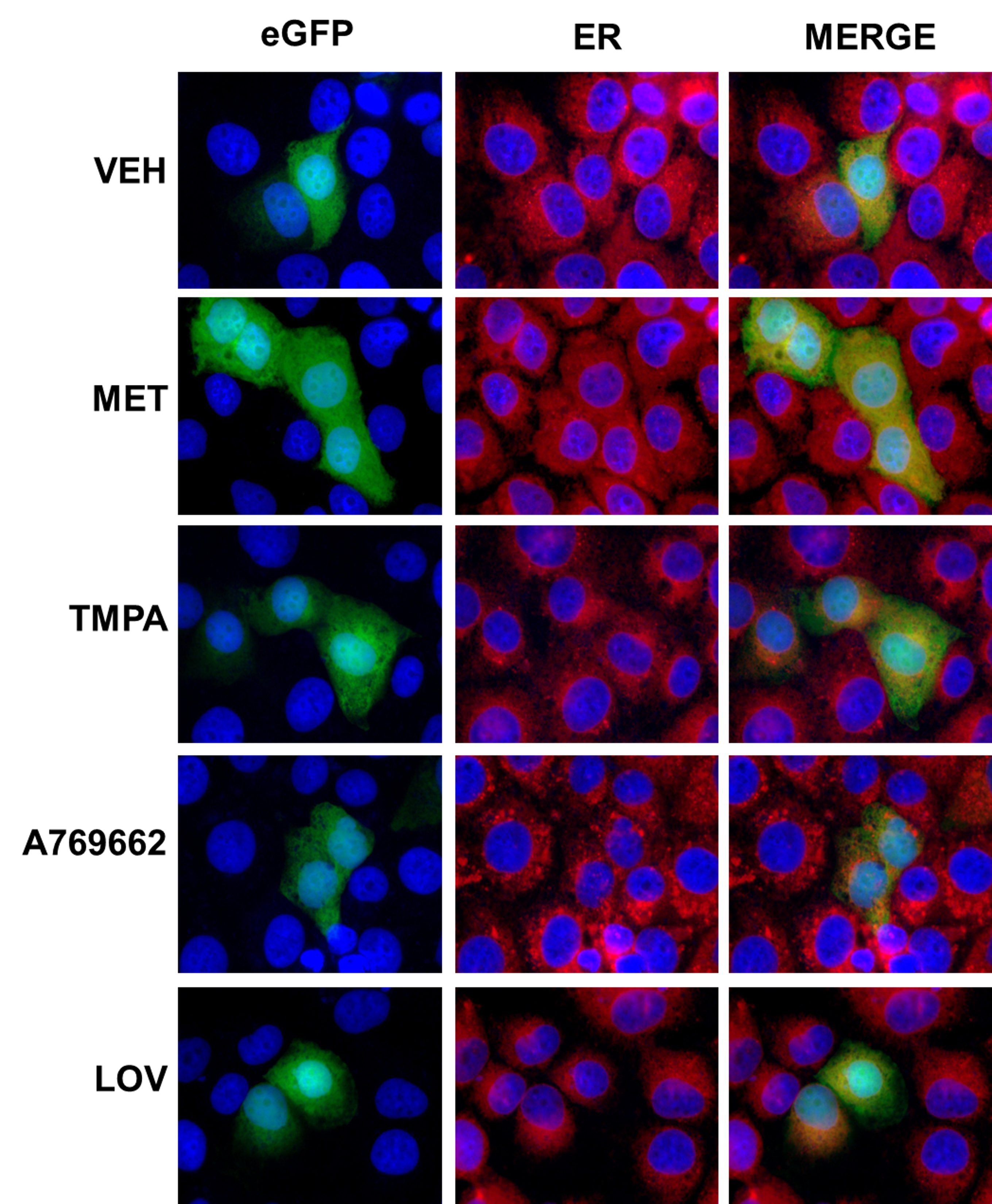

Supplement: S4 Fig — Huh7 cells were transfected with a plasmid codifying just eGFP [46] and 24 hours after, transfected cells were treated with DMSO 0.5% (VEH), 10 mM metformin (MET) 100 μM TMPA, 120 μM A-769662 and 50 μM lovastatin for 24 h. Distribution of eGFP (green) was analyzed by fluorescence microscopy, the endoplasmic reticulum (RE) was stained with (Concanavalin A Alexa fluor 594) (red).and Nuclei with dapi (blue). (TIF) [file ppat.1006257.s004.tif]

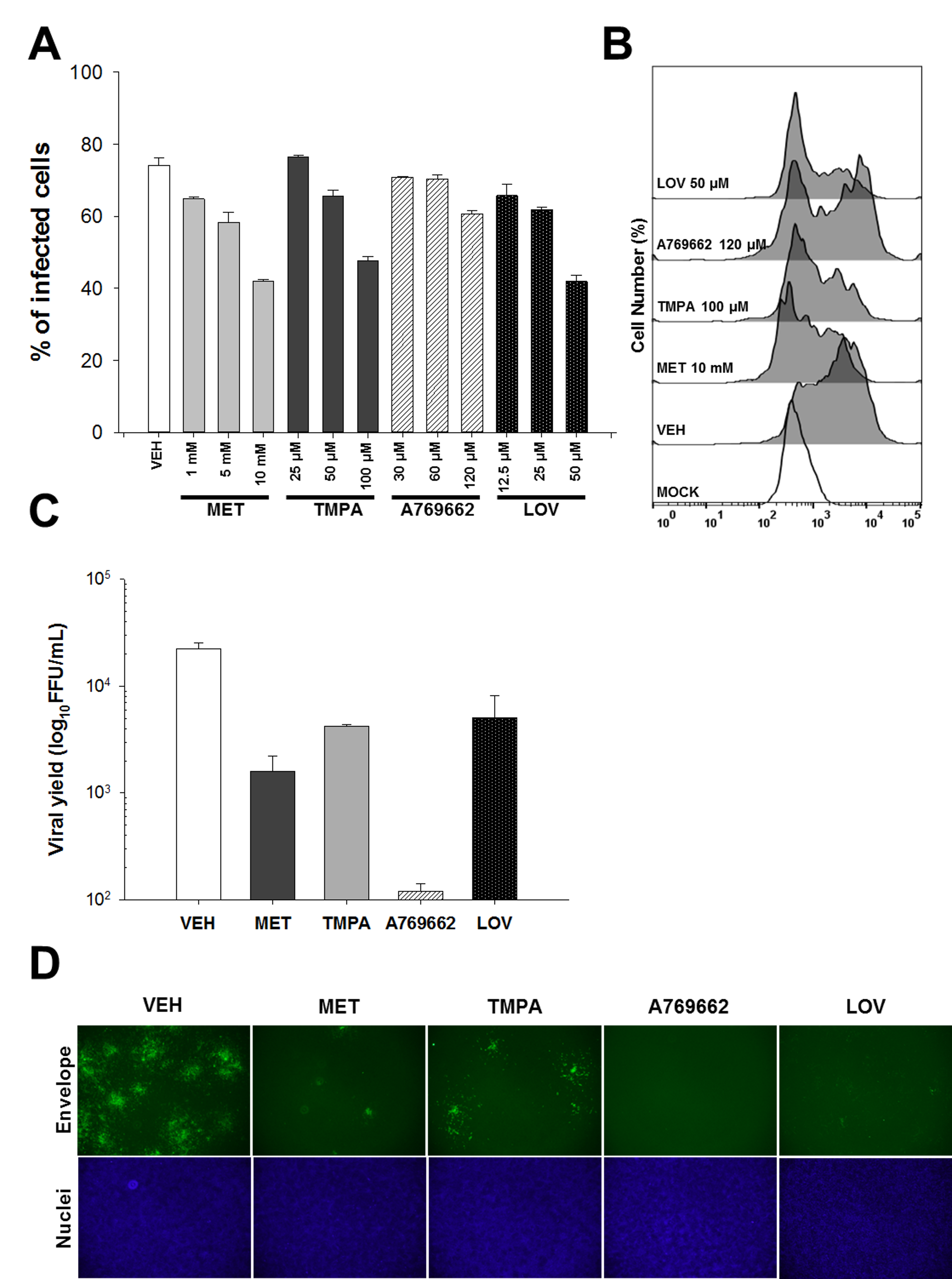

Supplement: S5 Fig — Huh7 cells were infected with DENV2 at a MOI 3 and treated for 24 h with metformin (MET 1 mM, 5 mM and 10 mM), TMPA (25, 50, 100 μM), A-769662 (30, 60, 120 μM) and lovastatin (LOV 12.5, 25, 50 mM). Percentage of infected cells was analyzed by flow cytometry (A) and histograms (B) depicting the mean fluorescence intensity are representatives of 3 independent experiment. Viral yield from supernatants was evaluated by foci assay and represented as log10FFU/mL of 3 independent experiments (C), immunofluorescences are representative of 3 experiments and (D) indicate the presence of foci (green) for each condition, nuclei (blue) were stained in order to demonstrate the monolayer integrity. (TIF) [file ppat.1006257.s005.tif]

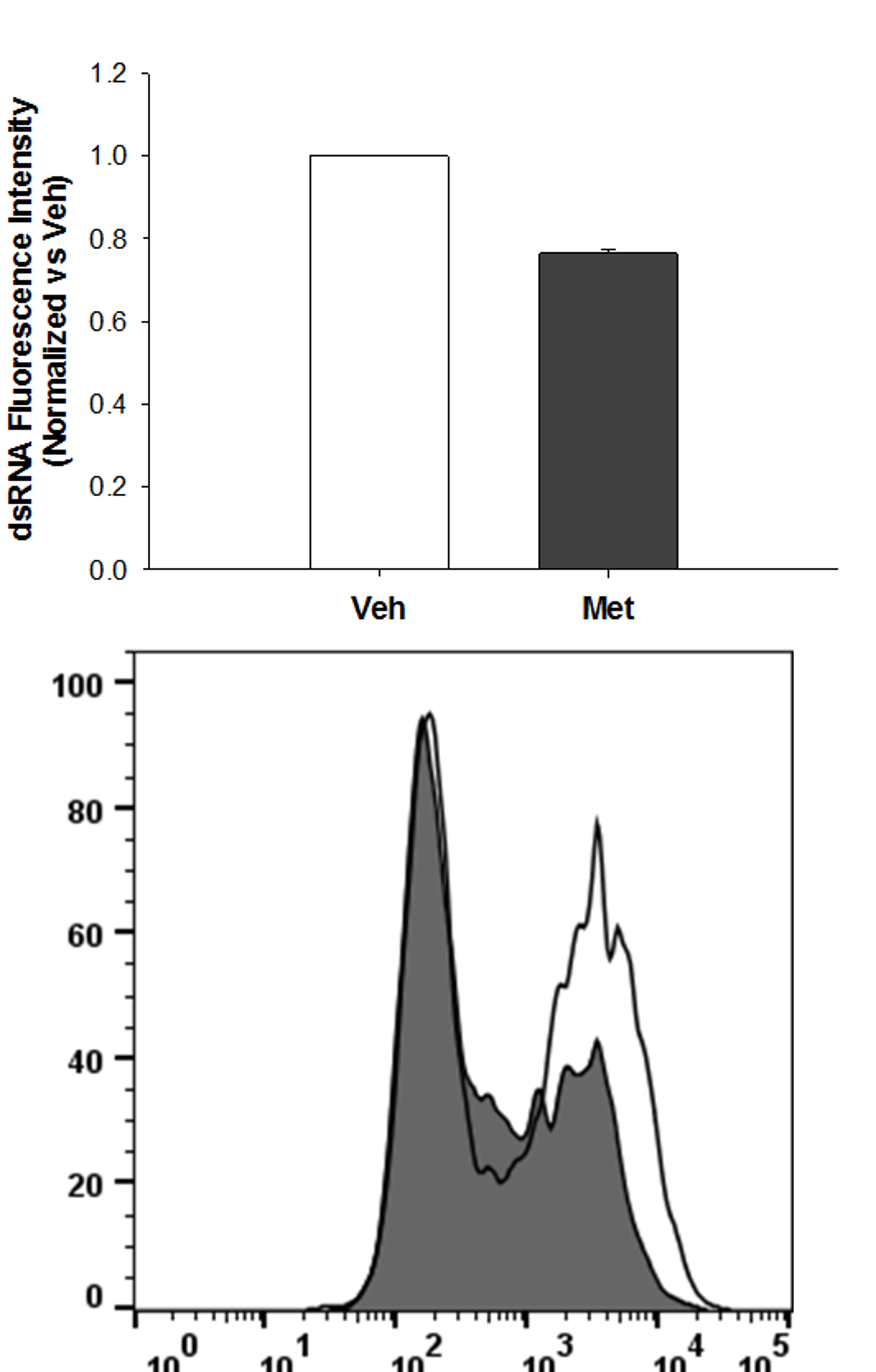

Supplement: S6 Fig — Huh7 cells were infected with DENV2 and treated with metformin10 mM or vehicle, and 24 hpi cells were fixed and stained for double strand RNA (antibody) and analyzed by flow cytometry. Graph represents the dsRNA mean fluorescence intensity from 3 experiments, Histograms are from a representative experiment. (TIF) [file ppat.1006257.s006.tif]

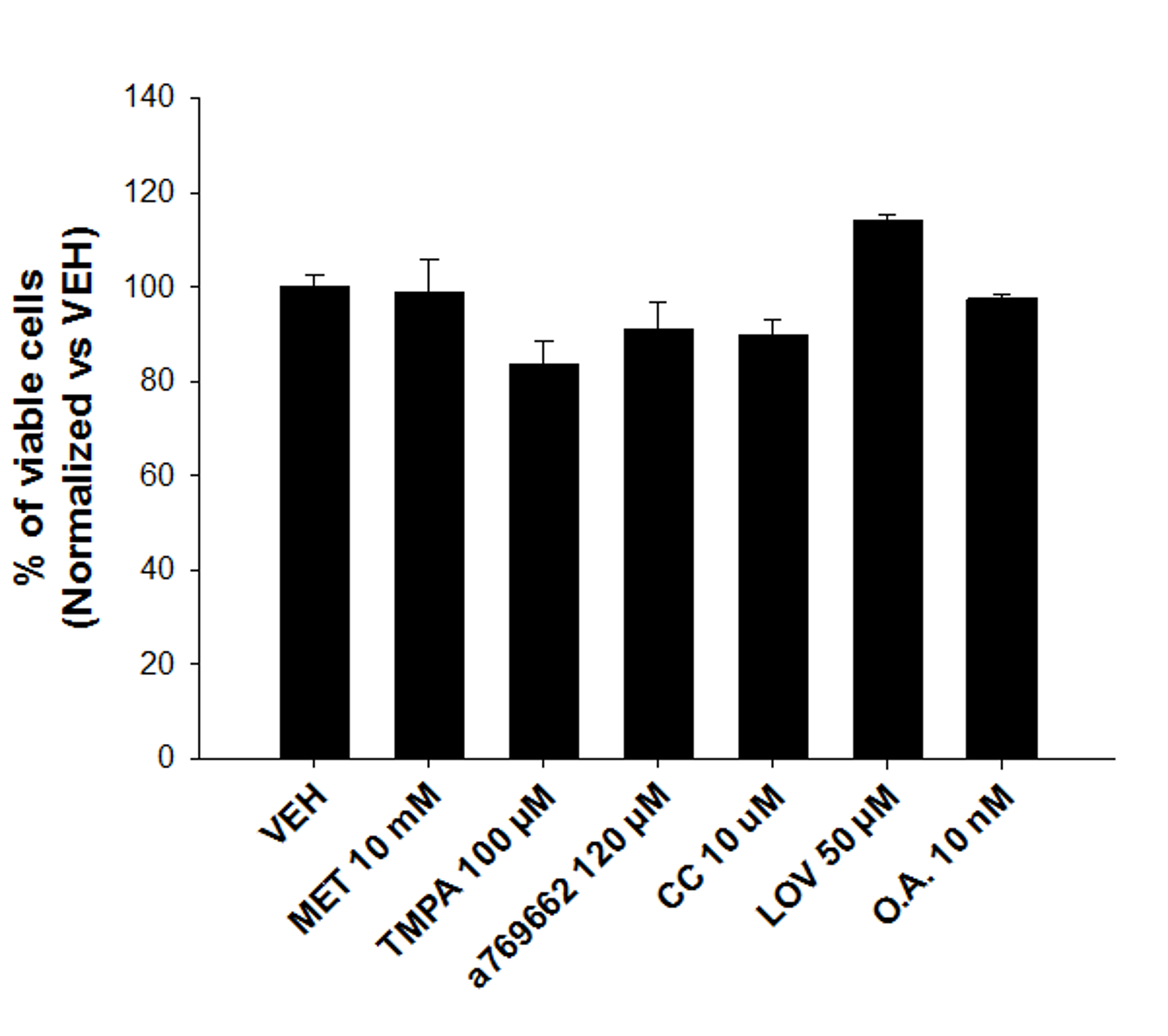

Supplement: S7 Fig — Cell viability was evaluated in Huh7 cells treated with drugs and concentrations indicated in the graph for 24 h using the cell proliferation assay CellTiter 96 AQueous (Promega). (TIF) [file ppat.1006257.s007.tif]

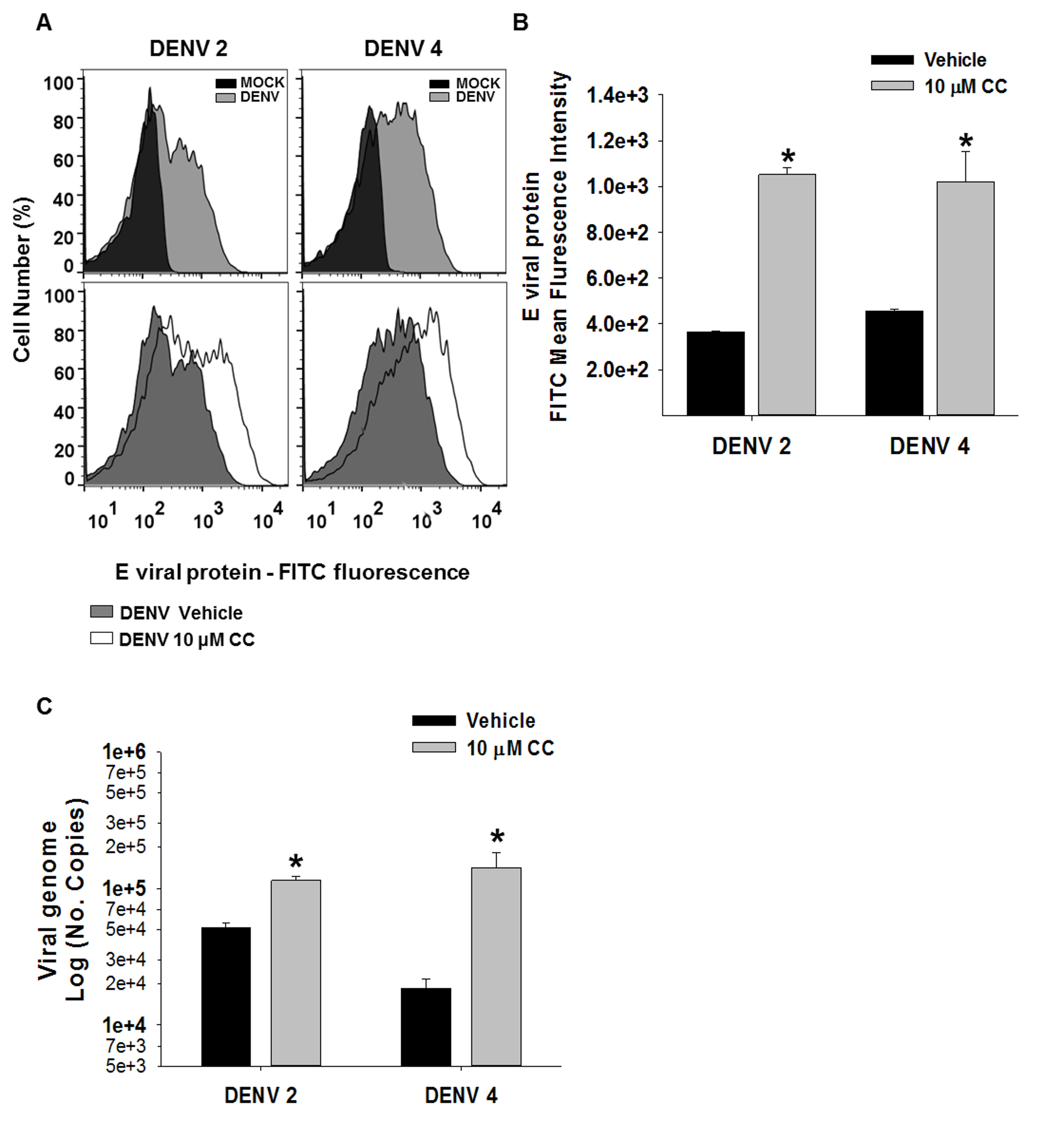

Supplement: S8 Fig — In A, The infection increase was evaluated by FACS using a mouse anti-E monoclonal antibody-4G2 to detect the E viral protein in Huh7 cells infected with Mock or DENV 2/4 (MOI 0.3), and treated with 10μM compound C (CC) or DMSO 0.5% (vehicle) for 24h. Upper histograms display the fluorescence of infected cells at 24h (gray filled histograms) respect to mock infected cells (dark histograms). Lower histograms show the fluorescence of DENV infected cells treated with CC (clear histograms) respect to vehicle-treated infected cells (dark histograms). B, The Mean Fluorescence intensity (MFI) for E viral protein is presented on Graphs. C, The number of viral genome copies of DENV 2/4 infected cells treated with DMSO 0.5% (vehicle) or 10μM CC for 24h was examined by qRT-PCR, and expressed as Log of No. Copies. Data are means ± S.E of n = 3 independent experiments realized by duplicated. * p<0.05 compared to vehicle-treated cells. (TIF) [file ppat.1006257.s008.tif]
